# Identification and characterization of transposable element *AhMITE1* in the genomes of cultivated and two wild peanuts

**DOI:** 10.1186/s12864-022-08732-0

**Published:** 2022-07-11

**Authors:** Yanyan Tang, Xiaoting Li, Changli Hu, Xiaochen Qiu, Jingjing Li, Xin Li, Hong Zhu, Jingshan Wang, Jiongming Sui, Lixian Qiao

**Affiliations:** College of Agronomy, Dry-Land Farming Technology Laboratory of Shandong Province, Key Laboratory of Qingdao Major Crop Germplasm Resource Innovation and Application, Qingdao Agricultural University, Qingdao, 266109 China

**Keywords:** MITEs, *AhMITE1*, Insertion preference, Genomes, Gene expression

## Abstract

**Background:**

The cultivated peanut (*Arachis hypogaea* L., AABB) is an allotetraploid hybrid between two diploid peanuts, *A. duranensis* (AA genome) and *A. ipaensis* (BB genome). Miniature inverted-repeat transposable elements (MITEs), some of which are known as active nonautonomous DNA transposons with high copy numbers, play important roles in genome evolution and diversification. *AhMITE1*, a member of the MITE family of transposons, but information on the peanut genomes is still limited. Here, we analyzed *AhMITE1*, *AuMITE1* and *ApMITE1* in the cultivated (*A. hypogaea*) and two wild peanut (*A. duranensis* and *A. ipaensis*) genomes.

**Results:**

The cultivated and the two wild peanut genomes harbored 142, 14 and 21 *AhMITE1*, *AuMITE1* and *ApMITE1* family members, respectively. These three family members exhibited highly conserved TIR sequences, and insertions preferentially occurred within 2 kb upstream and downstream of gene-coding and AT-rich regions. Phylogenetic and pairwise nucleotide diversity analysis showed that *AhMITE1* and *ApMITE1* family members have undergone one round of amplification bursts during the evolution of the peanut genome. PCR analyses were performed in 23 peanut varieties and demonstrated that *AhMITE1* is an active transposon and that hybridization or chemical mutagenesis can promote the mobilization of *AhMITE1*.

**Conclusions:**

*AhMITE1*, *AuMITE1* and *ApMITE1* family members were identified based on local BLAST search with MAK between the cultivated and the two wild peanut genomes. The phylogenetic, nucleotide diversity and variation copy numbers of *AhMITE1*, *AuMITE1* and *ApMITE1* members provides opportunities for investigating their roles during peanut evolution. These findings will contribute to knowledge on diversity of *AhMITE1*, provide information about the potential impact on the gene expression and promote the development of DNA markers in peanut.

**Supplementary Information:**

The online version contains supplementary material available at 10.1186/s12864-022-08732-0.

## Introduction

The cultivated peanut (*Arachis hypogaea* L.), also known as groundnut, is an allotetraploid (2n = 4x = 40) resulting from the hybridization of two wild diploids *A. duranensis* (AA genome) and *A. ipaensis* (BB genome). Peanut is an important oil crop with global production of 53.64 Mt (with shells) from an area of 31.57 Mha [1]. Mainland China boasts the largest peanut production of 17.99 Mt (with shells), compared with 9.95 Mt (with shells) in India [1]. Molecular breeding has played an important role in improving peanut varieties, which demands the development of genomic resources like linked markers for various traits. In peanut, genetic diversity is affected by polyploidization and is the source of lower levels of polymorphism than in diploid species [2, 3]. This limits the use of molecular marker-assisted breeding to enhance peanut production. Different types of markers should be developed and employed for diversity analysis with the completion of the assembly of peanut genome sequences [4, 5]. Among these, transposable elements (TEs) are the most abundant components of the genome and are used as genetic markers in molecular breeding.

TEs are major components of many plant and animal genomes and have been found in virtually all species investigated to date [6, 7]. *Ds* (*Dissociation*) was the first transposable element discovered by McClintock in maize [8]. TEs were once regarded as ‘selfish DNA’, but increasing evidence suggests that TEs are important in the generation of structure, evolution of genomes [9], and the regulation of gene function [10, 11]. In animals, Lynch et al. (2015) indicated that ancient TEs transformed the uterine regulatory landscape and transcriptome during the evolution of mammalian pregnancy [12]. Chuong et al. (2016) revealed that TEs, including endogenous retroviruses (ERVs), were involved in the regulation of essential immune functions [13]. TEs can be classified into class I and class II elements. The replication of class I elements, or retrotransposons and class II elements, DNA transposons, occurs through a ‘copy-and-paste’ mechanism and ‘cut-and-paste’ mechanism, respectively [14]. Class II elements are divided into two subclasses autonomous and nonautonomous [15], the difference is whether they contain transposases that mobilize TEs.

Miniature inverted-repeat terminal elements (MITEs) are short nonautonomous class II transposons that do not encode transposase [16–18]. MITEs possess a pair of conserved terminal inverted repeats (TIRs) and short target site duplications (TSDs) that are located outside of each TIR [19]. In many plant species including *Arabidopsis*, rice, maize, wheat, sorghum and peanut, MITEs have been reported and characterized [20–23]. MITE insertion has generated numerous polymorphisms, which have been exploited to develop molecular markers [24]. MITEs play an important role in gene regulation [25], as they preferentially accelerate the evolutionary process. In rice, the first active MITE *mPing* was identified in a slender glume mutant and also identified through genomic/computational analysis [18, 26, 27]. Tang et al. (2019) identified the active MITE *mJing* by analyzing a rice *high-tilling dwarf (htd*) rice mutant [28]. In maize, MITE insertion in the promotor of the gene *ZmNAC111* is associated with seedling drought tolerance [29]. Previous studies have improved that TEs can influence nearby gene expression. Such as, an 82 bp MITE insertion in the *ZmNAC111* promoter region was correlates with lower *ZmNAC111* expression in maize [29]. A *stowaway*-like MITE embedded in the 3'-UTR of the agronomically important gene *Ghd2* directly represses its protein synthesis, affecting grain number, plant height, and heading date in rice [30]. In peanut, an insertion of MITE in the *ahFAD2B* gene caused a frameshift, resulting in the high-oleate phenotype in Mycogen-Flavo and M2-225 mutants [22]. Shirasawa et al. (2012) found the *AhMITE1* in a gene for fatty-acid desaturase possessed excision activity [31]. Subsequently, 1039 *AhMITE1* markers were developed and used for mapping [32–34]. With the completion of peanut genome sequencing, TEs have been found to account for approximately 74% of the assembled genome sequence [4, 5]. Thus, the complete genome sequence offers excellent opportunities to study TEs and will contribute to our understanding of peanut diversity.

In this study, we used peanut sequencing data to determine the characteristics of *AhMITE1*. We identified the numbers of *AhMITE1* copies in cultivated peanut and its wild ancestors A. *duranensis* (AA genome) and A. *ipaensis* (BB genome). Following the genomic sequence, phylogenetic tree analyses of *AhMITE1* indicated that these elements clustered into five subfamilies. The genomic distribution and preferences of insertional loci were also investigated. The results improve understanding of the potential impact of *AhMITE1* on differentiation in cultivated and wild peanut species.

## Results

### Identification of *AhMITE1*s elements in peanut genomes

**Fig. 1:**
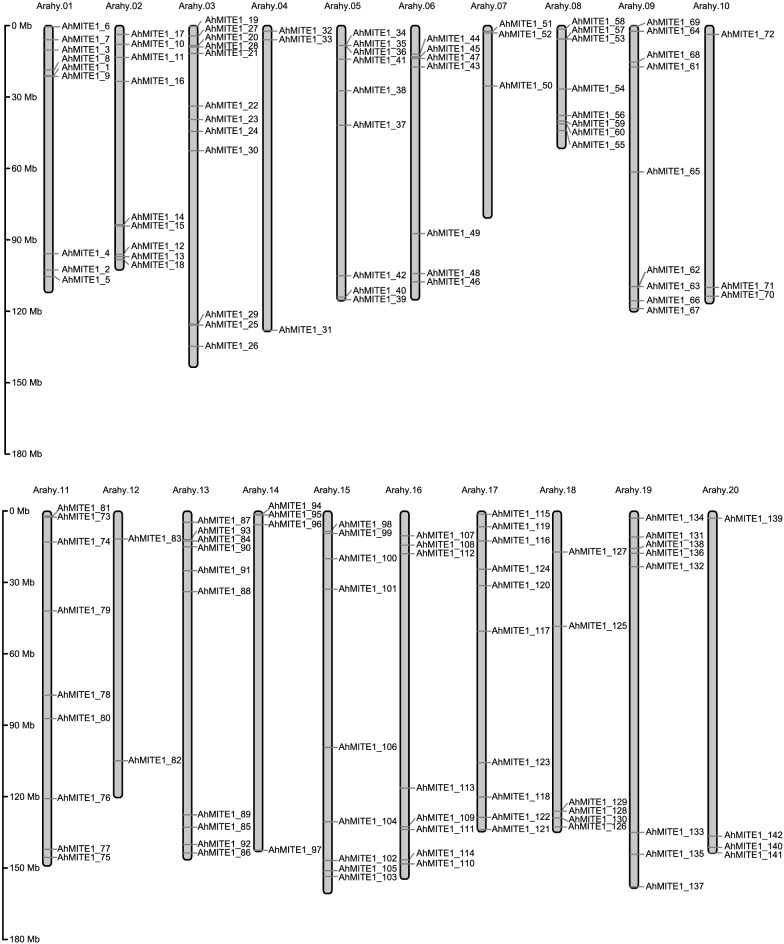
Chromosomal distribution of *AhMITE1* members in cultivated peanut *A. hypogaea* cv. Tifrunner. The chromosome numbers and sizes (Mb) are indicated at the top and left of each bar. Gray dashes represent the locations of each *AhMITE1* members

To investigate the *AhMITE1* family in the genomes of peanut, we performed a BLASTN analysis of genome to identify *AhMITE1* in the reference genome of *A. hypogaea* cv. Tifrunner and the two wild genomes of A. *duranensis* (AA genome) and A. *ipaensis* (BB genome). A previous study showed that the 205 bp *AhMITE1* includes a 9 bp target duplication (TSD) and 25 bp terminal inverted repeats (TIRs) [22, 31]. Finally, 142 copies of *AhMITE1* harboring the TSD and TIR sequences in the cultivated peanut genome were identified, and these copies shared over 90% similarity (Supplementary Table 1). According to the order of chromosomes 1–20 and the degree of similarity on the same chromosome, we named these *AhMITE1* elements *AhMITE1_1 to AhMITE1_142* (Supplementary Table 1). We used TBtools to investigate the positions of *AhMITE1* insertions in the peanut genome, and all *AhMITE1* elements were randomly distributed among the 20 chromosomes (Fig. 1 and Supplementary Table 1). However, these 142 *AhMITE1* elements were unevenly distributed and were preferentially located at the ends of chromosome arms rather than in the central portions of chromosome (Fig. 1).

Similarly, *AhMITE1* elements were also identified in the two wild genomes. Fourteen and 21 *AuMITE1* (*AuMITE1_1* to *AuMITE1_14*) and *ApMITE1* (*ApMITE1_1* to *ApMITE1_21*) elements were obtained from the *A. duranensis* and *A. ipaensis* genomes, respectively (Supplementary Table 2). The nomenclature for *AuMITE1* and *ApMITE1* was the same as in the cultivated peanut genome. In the *A. duranensis* genome, all chromosomes contained *AuMITE1* elements except for Aradu.A08 (Supplementary Fig. 1A). In the *A. ipaensis* genome, all chromosomes contained *ApMITE1* elements, and the chromosome Araip.B01 exhibited the highest number, with 4 *ApMITE1* elements (Supplementary Fig. 1B).

### Analysis of the TIR and TSD sequences of *AhMITE1* in the peanut genome

**Fig. 2:**
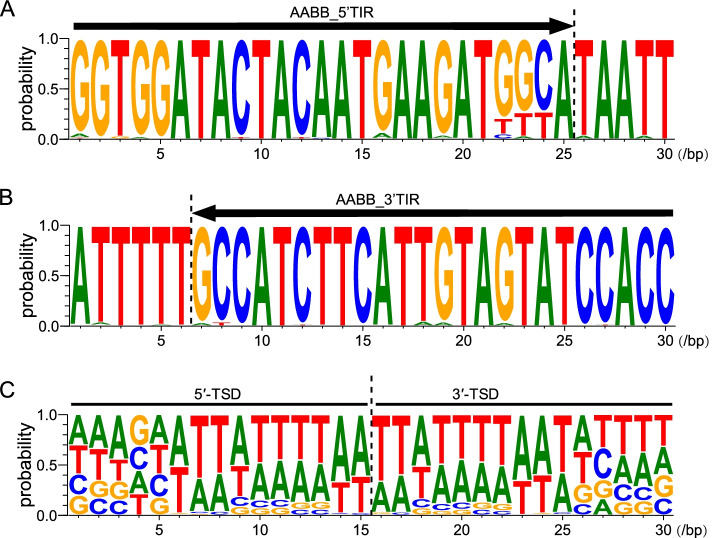
Conserved sequences of target site duplications (TSDs) and terminal inverted repeats (TIRs) in *AhMITE1* elements. **A** and **B** 5’ and 3’ TIRs of *AhMITE1* elements. **C** Conserved sequences of TSDs within the flanking regions. Black arrows and lines above the letters indicate the TSDs. The size of each letter indicates the frequency of the corresponding nucleotide

Through alignment of the TIR and TSD sequences of *AhMITE1* in the *A. hypogaea* genome, we found that most of the *AhMITE1* elements contained a 25 bp conserved TIR with the sequence 5’-GGTGGATACTACAATGAAGATGGCA-3’ (Fig. 2A and B). Almost all of the 9 bp TSD sequences in the 142 copies of *AhMITE1* were different, whereas most of them preferred the TTATTTTAA target site sequences (Fig. 2C).

In the two wild genomes, the TIR and TSD sequences of 14 *AuMITE1* and 21 *ApMITE1* elements were also analyzed. We found that the TIR and TSD sequences were similar to those in the *A. hypogaea* genome (Supplementary Fig. 2A, B, D and E). However, in the A. *duranensis* and A. *ipaensis* genomes, the TSD preferred AAAAAATAA/TAAAAATAA and TTAATAAAA/TTATAAAAA (Supplementary Fig. 2C and F).

### Analysis of *AhMITE1* sequence insertion sites in the peanut genome

**Fig. 3:**
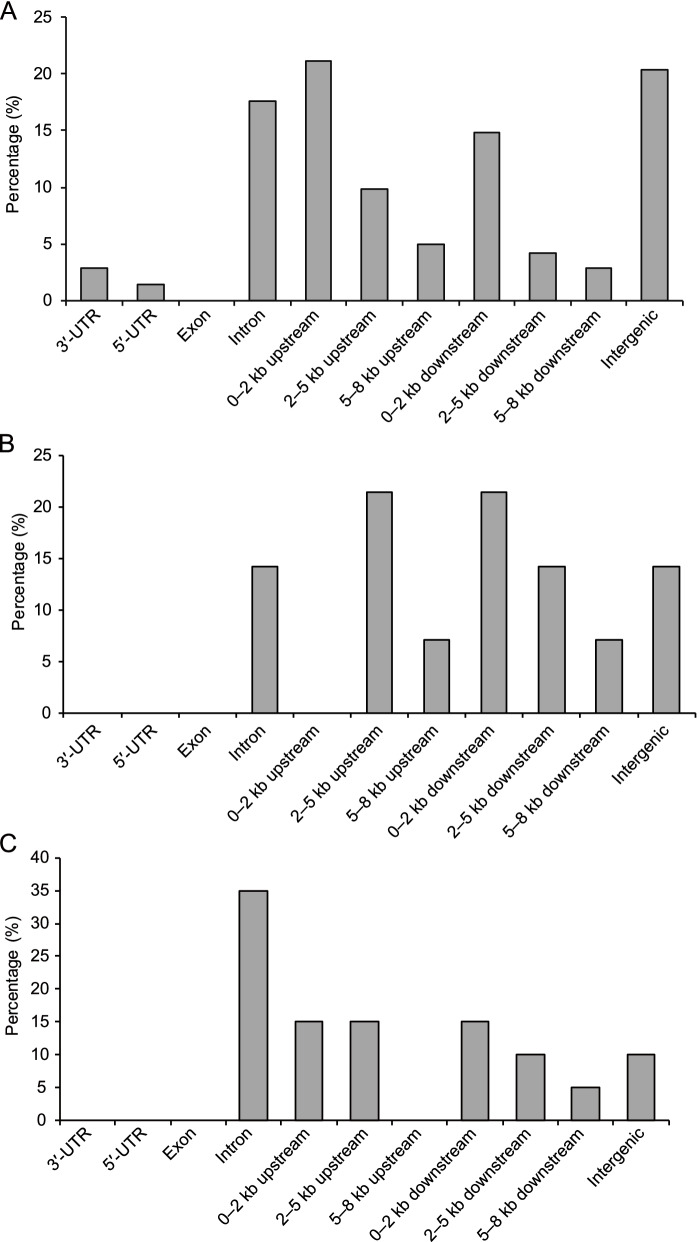
Insertion preferences of *AhMITE1* (A), *AuMITE1* (B) and *ApMITE1* (C) members in the genomes of *A. hypogaea* (cultivated peanut), *A. duranensis* and *A. ipaensis* genomes, respectively

Previous studies showed that approximately 45.83% (33 of 72) and 52.0% (133 of 256) of *mJing* and *mPing* insertions were within 2 kb and 3 kb of a coding regions, respectively [28, 35]. Investigation of the locations of 142 *AhMITE1* insertions in the *A. hypogaea* genome showed that 51 insertion events (35.92%) occurred 2 kb upstream and downstream of gene-coding regions, and 25 (17.61%) and 29 events (20.42%) were located in intergenic and intron regions, respectively (Fig. 3). In addition, 4 (2.82%) and 2 (1.41%) insertion events occurred in the 5’ and 3’-UTRs, respectively, no insertion events occurred in exonic regions (Fig. 3). Perhaps these insertion events that occurred in introns do not affect gene function. The location preference of *AhMITE1* insertions is consistent with the characteristics of *mJing* and *mPing* insertions in rice. The results suggested that the insertion of *AhMITE1* elements also occurred preferentially in promoter, intergenic and intron regions. As a control, 142 randomly selected sequences were preferentially inserted into intergenic, exonic and intronic regions rather than the promoter regions (Supplementary Fig. 3, Supplementary Table 5).

In the *A. duranensis* and A. *ipaensis* genomes, we found that the locations of insertion events were similar to those in *A. hypogaea*. In the *A. duranensis* genome, 2 (14.29%) and 3 (21.43%) insertion events occurred in introns and 2 kb upstream or downstream of gene-coding regions, respectively (Fig. 3B). In the *A. ipaensis* genome, 7 (33.33%) and 6 (28.57%) insertion events were located in introns and 2 kb upstream or downstream of gene-coding regions, respectively (Fig. 3C). Whether in the cultivated or the two wild peanut genomes, no insertion events occurred in exonic regions (Fig. 3).

### Analysis of GC content of *AhMITE1* sequences in the peanut genome

**Fig. 4:**
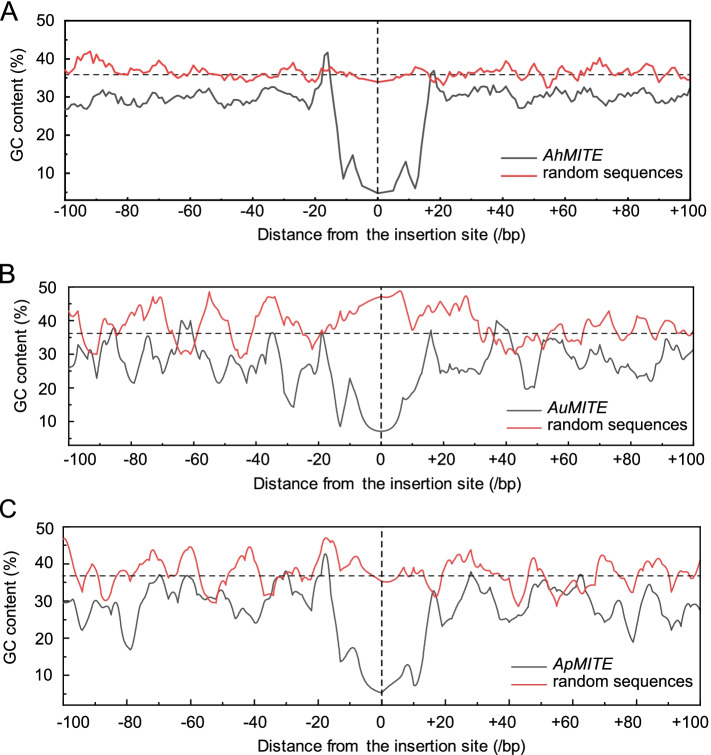
Sliding-window analysis of GC content within the flanking regions of *AhMITE1* (A), *AuMITE1* (B) and *ApMITE1* (C) members. Black lines indicate the average GC contents in the flanking sequences of *AhMITE1* (A), *AuMITE1* (B) and *ApMITE1* (C) members, respectively. Red lines indicate sequences from the *A. hypogaea*, *A. duranensis* and *A. ipaensis* genomes randomly selected as a controls. The origin on the *x*-axis indicates the insertion site of *AhMITE1* (A), *AuMITE1* (B) and *ApMITE1* (C) members

Previous studies have demonstrated that GC content averages 36.3589%, 36.0214% and 37.1722% in the *A. hypogaea*, *A. duranensis* and A. *ipaensis* genomes, respectively [4, 36, 37]. To determine whether the insertion of *AhMITE1* affects upstream and downstream sequences, sliding-window analysis of GC content at the *AhMITE1* insertions was conducted. One hundred upstream and downstream genome sequences close to the position of each *AhMITE1* insertion were examined, while 142 sequences 200 bp in length were randomly selected as a control (Supplementary Table 5). The results showed that the flanking regions near the *AhMITE1* insertion sites contained GC contents than those randomly selected 200 bp genome sequences in *A. hypogaea* (Fig. 4A). In addition, the GC contents of *AuMITE1* and *ApMITE1* were also lower than in the randomly selected sequences in the two wild peanut genomes (Fig. 4B, C, Supplementary Tables 6 and 7). Taken together, these results suggest that *AhMITE1*, *AuMITE1* and *ApMITE1* are all preferentially inserted into T/A rich regions in the peanut genome, which is consistent with the characteristics of other MITE family members [35].

### Sequence comparison of the *AhMITE1* family in the peanut genome

**Fig. 5:**
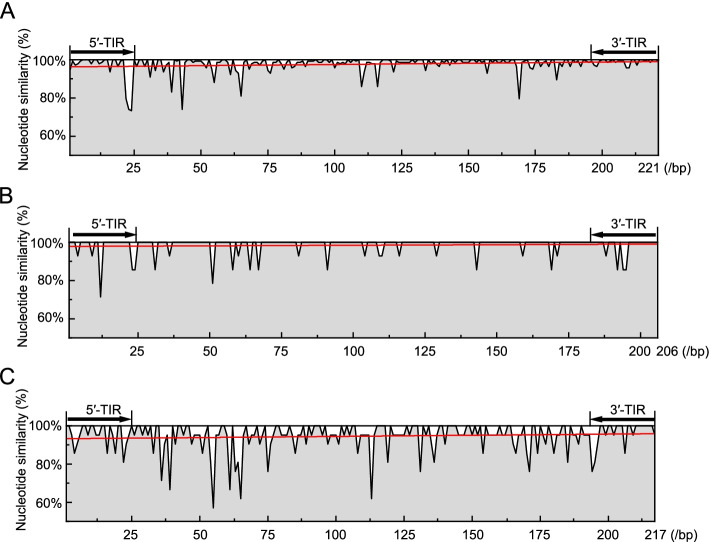
Analysis of sequence similarity among the 142 *AhMITE1* (A), 14 *AuMITE1* (B) and 21 *ApMITE1* (C) members in the genomes of *A. hypogaea* (cultivated genome), *A. duranensis* and *A. ipaensis*, respectively. Sequence similarity at each site was calculated based on sequence alignment via MUSCLE. The best fit for sequence similarity is represented by the red curve line

*AhMITE1* elements exhibited significant similarity in their internal sequences (Fig. 5A, Supplementary Fig. 4 and Supplementary Table 8). As with *AhMITE1* family, *AuMITE1* and *ApMITE1* members also exhibited high similarity in the two wild genomes (Supplementary Figs. 5 and 6). Comparing the similarities of *AhMITE1*, *AuMITE1* and *ApMITE1* sequences among the cultivated and two wild peanut genomes, we found that the *AhMITE1* family exhibited higher similarity in the *A. hypogaea* genome (Fig. 5A-C, Supplementary Table 9). We suspect that a one-time burst of *AhMITE1* family expansion occurred in the cultivated genome, similar to the expansion of the *mPing* family in rice.

### Phylogenetic analysis of the *AhMITE1* family in the peanut genome

**Fig. 6:**
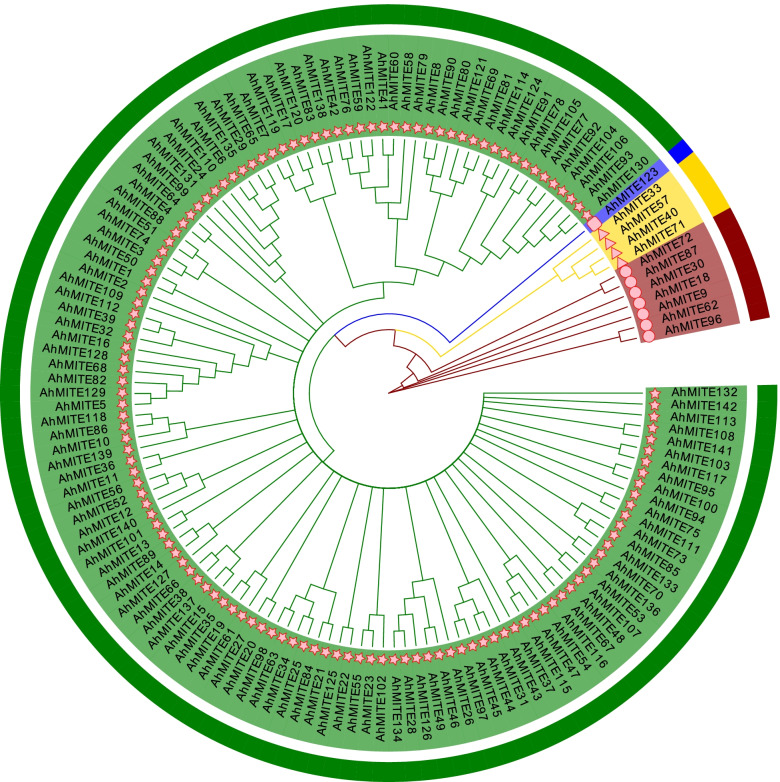
Phylogenetic tree comprising 142 *AhMITE1* elements from the *A. hypogaea* cv. Tifrunner genome. Green, blue, gold and red indicate Clade I, Clade II, Clade III and Clade IV, respectively, for 142 *AhMITE1* members

Phylogenetic analysis of *AhMITE1* family members separated these sequences into four clades (I, II, III, IV). Clade I was the largest branch with 120 members. Clade I and Clade II comprised 120 *AhMITE1* members and only one member, respectively, and were two subgroups from one branch (Fig. 6). Clade III and Clade IV contained 4 and 7 members, respectively (Fig. 6). In addition, three phylogenetic trees of 14 *AuMITE1* and 21 *ApMITE1* members from the *A. duranensis* and *A. ipaensis* genomes were also constructed, respectively. 14 *AuMITE1* members were separated into three clades (Supplementary Fig. 7A), and 21 *ApMITE1* members were divided into five clades (Supplementary Fig. 7B). However, in Supplementary Fig. 7C, 14 *AuMITE1* and 21 *ApMITE1* members were clustered into two Clades, 14 *AuMITE1s* were clustered into one Clade and 20 *ApMITE1s* were clustered into another Clade (Supplementary Fig. 7C), implying that the amplification of *AuMITE1* and *ApMITE1* members might occurred after the differentiation between AA and BB genomes.

**Fig. 7:**
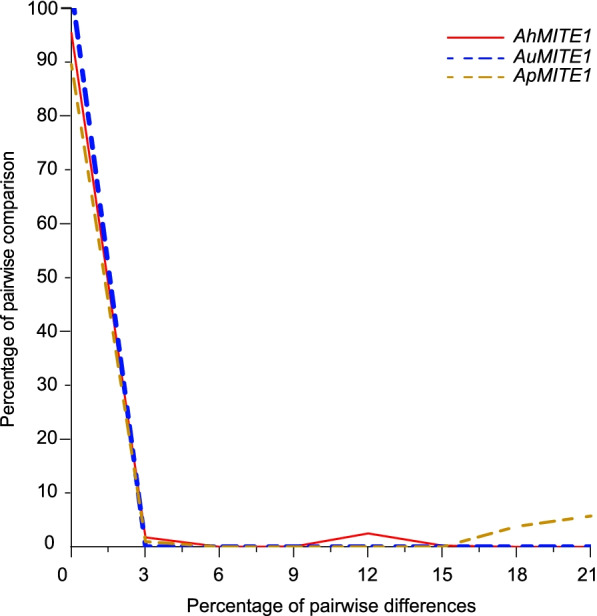
Frequency distribution of pairwise nucleotide diversity and analysis of sequence similarity among the *AhMITE1*, *AuMITE1* and *ApMITE1* members in the genomes of *A. hypogaea* (cultivated genome), *A. duranensis* and *A. ipaensis*, respectively. Red lines, blue dashes and gold dashes represent *AhMITE1*, *AuMITE1* and *ApMITE1* members in the genomes of *A. hypogaea*, *A. duranensis* and *A. ipaensis*, respectively

To investigate the amplification of *AhMITE1*, *AuMITE1* and *ApMITE1* members in the peanut genome, we calculated the pairwise nucleotide diversity. *AhMITE1*, *AuMITE1* and *ApMITE1* members exhibited similar peak distributions (Fig. 7). The histogram for the *AhMITE1*, *AhMITE1* and *AhMITE1* family has only the front face of a wave, centered at diversity = 0, the low nucleotide diversity indicated that these families are still under rapid amplification, like *mPing* family in rice [25, 35]. The *AhMITE1*, *AhMITE1* and *AhMITE1* families with unimodal distribution of pairwise nucleotide diversity have phylogenetic trees of a star shape (Supplementary Fig. 8).

### Validation of *AhMITE1* insertions in PCR amplification

**Fig. 8:**
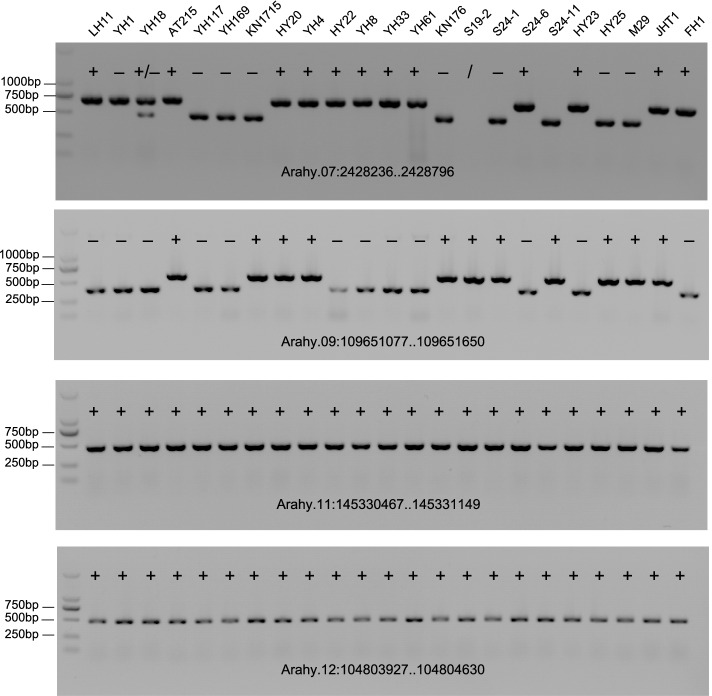
Validation of the presence of an *AhMITE1* insertion identified using PCR analysis in different varieties Validation of the presence of an *AhMITE1* insertion using locus-specific PCR analysis. Plus and minus represent the presence and absence of the *AhMITE1* insertion, respectively.

There are differences in the numbers of *AhMITE1* members copies among cultivated peanut varieties. Based on the polymorphism of 142 *AhMITE1* members in cultivated peanut, 12 *AhMITE1* insertions on the 20 chromosomes were selected and verified by PCR using 23 varieties (Supplementary Table 10). These different accessions of peanut may be polymorphic in their presence or absence of *AhMITE1* (Fig. 8 and Supplementary Fig. 9). For example, there are no *AhMITE1* insertions in the peanut varieties LH11 and YH1, and a homozygous insertion occurred in YH18, which is the hybrid progeny of LH11 and YH1 at the location Arahy.18:48,326,860.0.48327510 (Supplementary Fig. 9). In the mutant lines S19-2, S24-1, S24-6 and S24-11 derived from parental HTY22 by chemical mutagenesis, a homozygous *AhMITE1* insertion were present at Arahy.07:2,428,236.0.2428796, while S24-1 and S24-11 showed no insertions. At the location Arahy.09:109,651,077.0.109651650, HY22 had no insertion of *AhMITE1*, however, insertion occurred in the mutant lines S19-2, S24-1 and S24-11. These results indicated that *AhMITE1* is an active transposon and that hybridization or chemical mutagenesis can promote the mobilization of *AhMITE1*. At the locations Arahy.11:145,330,467.0.145331149 and Arahy.12:104,803,927.0.104804630, *AhMITE1* insertions were detected in all the 23 peanut varieties. Thus, the presence or absence of *AhMITE1* in different varieties could be developed into molecular markers and utilized in peanut species.

In this study, the effect of *AhMITEs* on the expression of genes was assessed and compared it between cultivated species. We found that *AhMITE1* insertions occurred in some cultivated species, and some did not. We compared the expression levels of adjacent genes (*Arahy.7UQ7HQ* and *Arahy.8H8JUA*), and found that *AhMITEs* insertion into the promoter of *Arahy.7UQ7HQ* (Arahy.07:2,429,614.0.2,431,796) and *Arahy.8H8JUA* (Arahy.09:109,642,643.0.109,652,456) would reduce the genes expression level compared with no *AhMITEs* insertion (Supplementary Fig. 10). Our findings suggest that the insertion of *AhMITEs* can influence nearby gene expression.

## Discussion

In the present study, 124, 14 and 21 *AhMITE1*, *AuMITE1* and *ApMITE1* members were identified from the *A. hypogaea*, *A. duranensis* and *A. ipaensis* genomes, respectively. The representative 205 bp *AhMITE1* sequence was AT-rich, with 9 bp TSDs and 25 bp TIRs, similar to those previously reported for peanut [22, 31]. Comparison of TIR and TSD sequences revealed complete conservation of TIRs, but not TSDs. *AhMITE1* preferentially inserted into the 2 kb upstream and downstream sequences of coding regions, especially T/A-rich regions, in the peanut genome. Pairwise nucleotide difference analyses demonstrated that *AhMITE1* members underwent one round of amplification during peanut evolution.

Genome-wide analysis revealed that different copy numbers of *AhMITE1*, *AhMITE1* and *ApMITE1* were present in the genomes of cultivated and the two wild peanut genomes. The copy number for *AhMITE1* was far greater than those for *AuMITE1* and *ApMITE1*. Differences in the copy numbers of *AhMITE1*, *AhMITE1* and *ApMITE1* between cultivated and wild peanut might also affect amplification and selection during peanut evolution. In the *A. hypogaea* genome, the copy number of *AhMITE1* identified was 142 members, which is significantly higher than those in the *A. duranensis* (14) and *A. ipaensis* (21) genomes. DNA methylation, generally considered to be a heritable epigenetic modification that functions in silencing TEs, plays roles in the maintenance of genome stability, genetic imprinting and the regulation of gene expression [38–41]. Methylation was lower in the A subgenome than in the B subgenome [5]. Thus, there may be other reasons, such as chromosome structure, chromosome inversions and small RNAs, that caused the copy number differences in the AA and BB genomes. These differences in copy number suggest that the *AhMITE1*, *AuMITE1* and *ApMITE1* members maintained transpositional activation after the differentiation of cultivated and wild peanut.

The *AhMITE1* members preferentially inserted within 2 kb upstream or downstream of the gene-coding and genetic regions, and no insertions occurred in exons of the gene regions. Previous studies have also demonstrated that the insertion of both *mJing* and *mPing* elements were preferentially in the flanking region of the gene [28]. In addition, 142 *AhMITE1* elements were preferentially located at the ends of chromosome arms rather than in the central regions of chromosomes. Bertioli et al. (2019) demonstrated that gene densities are highest in distal chromosome regions and DNA transposons are more frequent in euchromatic arms in peanut [4]. Taken together, we suspect that this may be the reason why *AhMITE1* elements preferentially inserted into the promoters of gene-coding regions and were located in the distal chromosome regions. As for MITEs, the density of genes is higher in distal regions of chromosomes.

The nucleotide diversity among *AhMITE1* elements may reflect the amplification characteristics during peanut evolution. The pairwise nucleotide diversity of *AhMITE1*, *AuMITE1* and *ApMITE1* members in cultivated and wild peanut revealed the occurrence of different amplification pattern bursts during peanut evolutionary history, which is similar to patterns observed in the MITE families in rice [25]. The pairwise nucleotide diversity of *AuMITE1* is lower than that of *AhMITE1* and *ApMITE1* members indicating that *AuMITE1* amplification occurred before *AhMITE1* arose during peanut genome evolution. Therefore, both phylogenetic and pairwise nucleotide difference analyses demonstrated that *AhMITE1*, *AhMITE1* and *AhMITE1* families have experienced one-time burst expansion during peanut evolution. In Supplementary Fig. 7C, 14 *AuMITE1* and 21 *ApMITE1* members were clustered into two Clades, 14 *AuMITE1s* were clustered into one Clade and 20 *ApMITE1s* were clustered into another Clade (Supplementary Fig. 7C). These results suggested that the amplification of *AuMITE1* and *ApMITE1* members possibly occurred later than the differentiation of the two wild peanuts.

In our study, common and high-oleic-acid peanut varieties, such as HY20 and YH117, differed in *AhMITE1* insertions (Fig. 8 and Supplementary Fig. 9). This phenomenon also occurs in peanut varieties from different countries (Fig. 8 and Supplementary Fig. 9), such as AT215 and other varieties (Fig. 8 and Supplementary Fig. 9). In addition, there were no *AhMITE1* insertions in the peanut varieties LH11 and YH1, and a homozygous insertion occurred in YH18, which is the hybrid progeny of LH11 and YH1, at the location Arahy.18: 48,326,860.0.48327510 (Supplementary Fig. 9). The detected de novo insertions in the hybrid progeny and mutant lines suggested that the mobilization of *AhMITE1* might be induced by hybridization. Therefore, this study provides evidence to support the hypothesis that transposons are activated by "genome shock" due to plant-wide hybridization [41].

In peanut, DNA polymorphism is very low due to the narrow genetic diversity, which limits the development of markers [42–44]. The very limited genotypic polymorphism despite enormous phenotypic differences among peanut genotypes signifies the requirement for a large number of markers [45]. In many studies, have showed that different patterns of MITE insertions in germplasms or individuals have been used as DNA markers in plants [28, 46–48]. Thus, the development of *AhMITE1* members as DNA markers is worth considering. Targeted high-throughput sequencing is an efficient method for identifying the insertion positions of specific MITE members in the genomes of different peanut varieties [28]. Finally, these polymorphisms involving the presence or absence of *AhMITE1* at loci could be used to develop DNA markers. The variation in *AhMITE1* copy numbers contributes to our understanding of peanut diversity. Therefore, our identification of *AhMITE1*, *AuMITE1* and *ApMITE1* members provides opportunities for investigating their roles during peanut evolution.

## Materials and methods

### Plant materials

To validate *AhMITE1* insertions in PCR amplification, 23 peanut samples including common varieties (LH11, HY20, HY22, HY23, HY25, YH1, YH4, YH8, JHT1, FH1), high-oleic acid varieties (AT215, YH18, YH33, YH117, YH61, YH169, KN1715, KN176), and mutant lines (S19-2, S24-1, S24-6, S24-11, M29) were used. In addition, seeds of the wild peanut species two wild diploids *A. duranensis* and *A. ipaensis* were obtained from the USDA or NCSU germplasm collections. Peanut seeds were germinated in distilled water and then planted in pots filled with matrix media. All peanut plants were grown in a greenhouse, and the growth conditions were set as 16 h/8 h of light/dark at 24 °C. For the field trials, peanut plants were grown in a field in Qingdao (36.04°N, 120.19°E), China.

### Identification of peanut *AhMITE1*, *AuMITE1 *and *ApMITE1* members in the peanut genome

Both the cultivated (*A. hypogaea* cv. Tifrunner) and two wild peanut (*A. duranensis* and *A. ipaensis*) reference genome sequences were downloaded from the peanut genome database (PeanutBase, https://peanutbase.org/data/public/). A local BLAST search with MAK was performed using the 205 bp *AhMITE1* as a query [49]. As a result, many elements were aligned. Then, the following settings were used length = 205 bp, E-value < 10^–71^ and the similarity > 90.00%. The target sequences were discarded if they did not satisfythese conditions. In total, 142, 14 and 21 full-length *AhMITE1*, *AuMITE1* and *ApMITE1* members were discovered, respectively. Information about all of the *AhMITE1*, *AuMITE1* and *ApMITE1* members is provided in Supplementary Tables 1 and 2.

### Analyses of the chromosomal distribution of *AhMITE1*,* AuMITE1 *and *ApMITE1* members

All *AhMITE1*, *AuMITE1* and *ApMITE1* elements were mapped to chromosomes based on physical location information from the peanut genome database (*A. hypogaea*, *A. duranensis* and *A. ipaensis* genome sequences) using TBtools (Graphics/Show Genes on Chromosome/Gene Location Visualize (advanced)) [50].

### Multiple sequence alignment of *AhMITE1*,* AuMITE1 *and *ApMITE1* members

A multiple sequence alignment of *AhMITE1*, *AuMITE1* and *ApMITE1* members was performed using CLUSTALW with default parameters [51]. TSD and TIR sequences was truncated from the 142, 14 and 21 members of *AhMITE1*, *AuMITE1* and *ApMITE1*. Nine bp and 21 bp TSD and TIR sequences of *AhMITE1*, *AuMITE1* and *ApMITE1* were depicted using WebLogo 3.0, respectively [52].

### Insertion preferences of *AhMITE1*,* AuMITE1 *and *ApMITE1* members

To analyze the insertion preferences of *AhMITE1*, *AuMITE1* and *ApMITE1* members, 142 *AhMITE1*, 14 *AuMITE1* and 21 *ApMITE1* insertion sites were aligned with the *A. hypogaea*, *A. duranensis* and *A. ipaensis* genomes (https://peanutbase.org/gbrowse_peanut1.0), respectively. Based on each distance between an insertion site and the annotated gene, the position of insertion was categorized as being in the 3’-UTR, 5’-UTR, exon or intron, upstream or downstream of the annotated gene coding region. If the distance between the insertion site and the annotated gene exceeded 8 kb (> 8 kb), the insertion site was categorized as being in an intergenic region.

### Analysis of GC content

Sample 100 bp upstream and downstream sequences with TSDs of 142 *AhMITE1*, 14 *AuMITE1* and 21 *ApMITE1* members were extracted from the genomes of *A. hypogaea*, *A. duranensis* and *A. ipaensis*, respectively. In addition, 142, 12 and 21 of sequences 205 bp in length were randomly selected with TBtools (Sequence Toolkit/Fasta Tools/Fasta Extract (Basic)) from the *A. hypogaea*, *A. duranensis* and *A. ipaensis* genomes, respectively [50]. A PERL script (Supplementary Table 11) was used to calculate the GC content in each 5-bp sliding-window at a 1 bp increment.

### Estimation of pairwise diversity

To investigate the amplification of *AhMITE1*, *AuMITE1* and *ApMITE1* members in the peanut genome, we calculated the pairwise nucleotide diversity for each species. Pairwise nucleotide diversity among *AhMITE1*, *AuMITE1* and *ApMITE1* elements in the *A. hypogaea*, *A. duranensis* and *A. ipaensis* genomes was calculated using a PERL script and MEGA [53]. If there was a gap in the sequence alignment, each gap was considered to be a single mismatch. The obtained pairwise nucleotide diversity values were plotted with OriginPro 2018 software. The frequency distribution of pairwise nucleotide diversity was used to describe the amplification patterns during peanut genome evolution.

### Phylogenetic analysis

In total, 142 *AhMITE1*, 14 *AuMITE1* and 21 *ApMITE1* members from *A. hypogaea*, *A. duranensis* and *A. ipaensis* genomes were aligned using MUSCLE, respectively [54]. The alignment results were used to construct a phylogenetic tree in MEGA 6.0 by the neighbor-joining (NJ) method with a bootstrap test of 1000 replications [38], and visually enhanced by Evolview [55].

### PCR amplification of *AhMITE1* insertion sites

To validate the *AhMITE1* insertions in the cultivated peanut genomes, PCR primers were designed based on the flanking sequences of the *AhMITE1* insertion. PCR amplifications were performed using 10 ~ 50 ng peanut genomic DNA in a 5 μL reaction volumes containing 0.2 µL of LA Taq® DNA polymerase (1 U), 0.2 µL of dNTP (200 mM), 1 µL of 10 × PCR buffer and 1 µL each of forward and reverse primers (30 μM). The PCR protocol was performed with an initial denaturation at 94 °C for 5 min, followed by 35 cycles of 94 °C for 30 s, 50 °C for 30 s and 72 °C for 45 s, and a final extension at 72 °C for 10 min. The products were separated using polyacrylamide gel electrophoresis (PAGE) in 1% (w/v) agarose gels to distinguish the presence or absence polymorphisms of *AhMITE1* insertions in the cultivated peanut genomes.

### RNA extraction and RT-PCR

Total RNA from the leaves was extracted using TRIzol reagent (Thermo Fisher Scientific) and purified using a Qiagen RNeasy Kit (Qiagen, Hilden, Nordrhein-Westfalen, Germany) according to the manufacturer’s instructions. First-strand cDNAs were synthesized using SuperScript RT Kit (Thermo Fisher Scientific) with an oligo(dT)_12–18_ primer. RT-PCR was performed using a CFX96 real-time system (Bio-Rad, Hercules, CA) and the peanut expressing gene *U6* was used as the internal control to normalize the gene expression data. Relative expression levels were calculated from three biological replicates. qRT-PCR was performed with a CFX96 real-time system (Bio-Rad, Hercules, CA). All reactions were performed with three biological replicates. Statistical analysis and sample comparisons were performed using the relative quantification method (2^−ΔΔCT^).

### Primers

The primers used in this study are listed in Supplementary Table 12.

## Supplementary Information

**Additional file 1:** **Supplementary fig 1.** 

**Additional file 2:** **Supplementary fig 2.**

**Additional file 3: Supplementary fig 3.**

**Additional file 4: Supplementary fig 4.**

**Additional file 5: Supplementary fig 5.**

**Additional file 6: Supplementary fig 6.**

**Additional file 7: Supplementary fig 7.**

**Additional file 8: Supplementary fig 8.**

**Additional file 9: Supplementary fig 9.**

**Additional file 10: Supplementary fig 10.**

**Additional file 11: Supplementary table 1.**

**Additional file 12: Supplementary table 2.** 

**Additional file 13: Supplementary table 3.**

**Additional file 14: Supplementary table 4. **

**Additional file 15:** **Supplementary table 5.** 

**Additional file 16: Supplementary table 6.** 

**Additional file 17: Supplementary table 7.** 

**Additional file 18: Supplementary table 8.**

**Additional file 19: Supplementary table 9.** 

**Additional file 20: Supplementary table 10.**

**Additional file 21: Supplementary table 11.**

**Additional file 22: Supplementary table 12.**

## Data Availability

Assembled whole genomic sequence of *Arachis hypogaea*. L was accessed from NCBI repository (https://ftp.ncbi.nlm.nih.gov/genomes/genbank/plant/Arachis_hypogaea/all_assembly_versions/GCA_003086295.2_arahy.Tifrunner.gnm1.KYV3/), with WGS project PIVG01, Bio project number PRJNA419393 and Bio sample number SAMN08051159. Assembled whole genomic sequence of *Arachis duranensis* was accessed from NCBI repository (https://ftp.ncbi.nlm.nih.gov/genomes/genbank/plant/Arachis_duranensis/all_assembly_versions/GCA_000817695.3_aradu.V14167.gnm2.J7QH/), with WGS project JQIN01, Bio project number PRJNA258023 and Bio sample number SAMN02982871. Assembled whole genomic sequence of *Arachis ipaensis* was accessed from NCBI repository (https://ftp.ncbi.nlm.nih.gov/genomes/genbank/plant/Arachis_ipaensis/all_assembly_versions/GCA_000816755.2_Araip1.1/), with WGS project JQIO01, Bio project number PRJNA258025 and Bio sample number SAMN02982874. The NCBI accession numbers PRJDB5785 of *AhMITE1* used in this study. All data generated or analyzed during this study are included in this published article (and its supplementary information files).
