# Supplementary material for: Identification and characterization of transposable element AhMITE1 in the genomes of cultivated and two wild peanuts
Source: BMC Genomics. 2022 Jul 11;23:500. doi: 10.1186/s12864-022-08732-0 (PMC9277781; doi:10.1186/s12864-022-08732-0)
Supplement: Supplementary file 1 — Additional file 1: Supplementary fig 1. [file 12864_2022_8732_MOESM1_ESM.pdf]

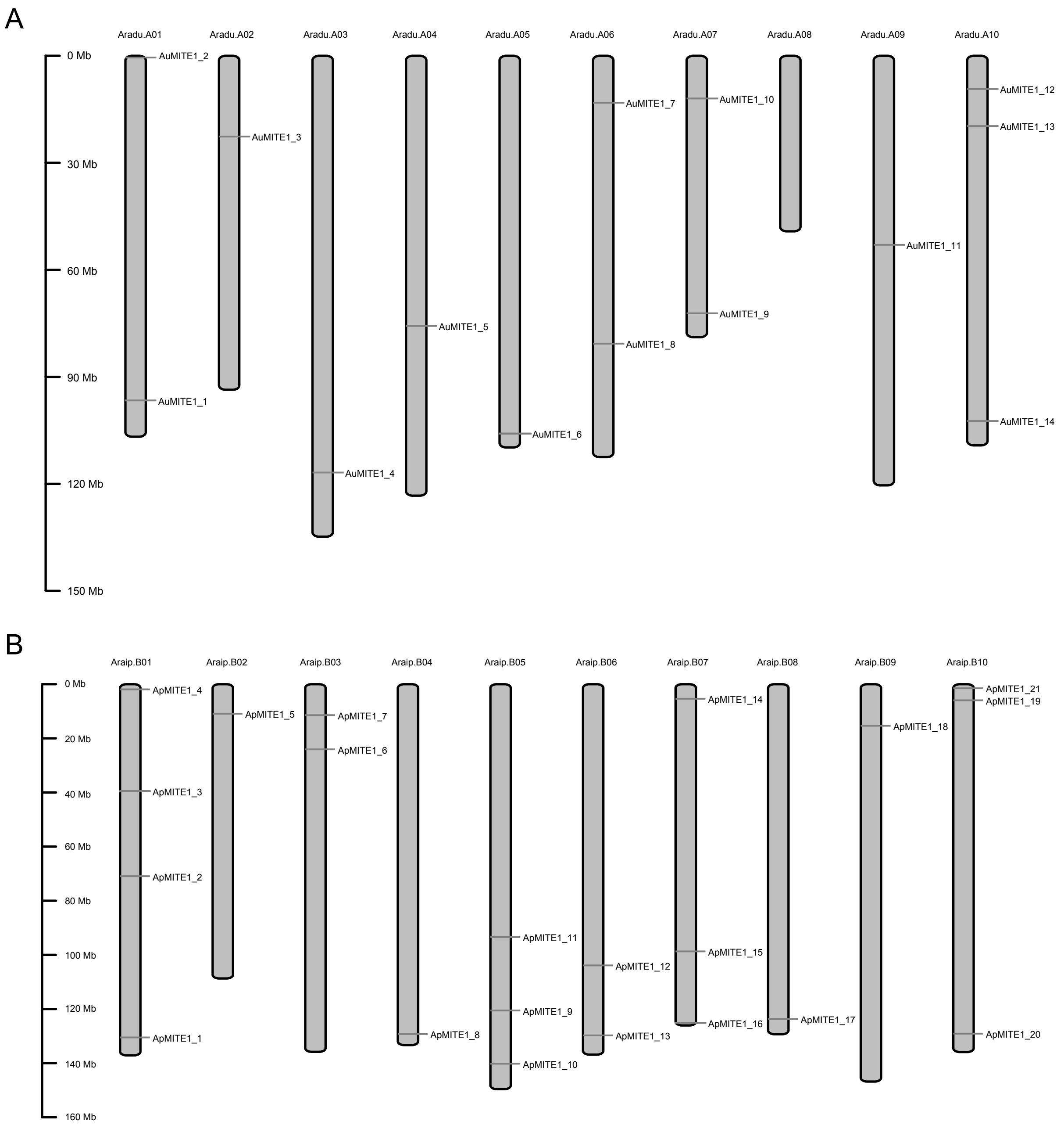

Supplementary Fig. 1 Chromosomal distribution of *AuMITE1* (A) and *ApMITE1* (B) members inthe two wild genomes *A. duranensis* and *A. ipaensis*, respectively. The chromosome numbers and sizes (Mb) are indicated at the top and left of each bar. Gray dashes represent the locations of *AuMITE1* (A) and *ApMITE1* (B) members.
