# Supplementary material for: Identification and characterization of transposable element AhMITE1 in the genomes of cultivated and two wild peanuts
Source: BMC Genomics. 2022 Jul 11;23:500. doi: 10.1186/s12864-022-08732-0 (PMC9277781; doi:10.1186/s12864-022-08732-0)
Supplement: Supplementary file 2 — Additional file 2: Supplementary fig 2. [file 12864_2022_8732_MOESM2_ESM.pdf]

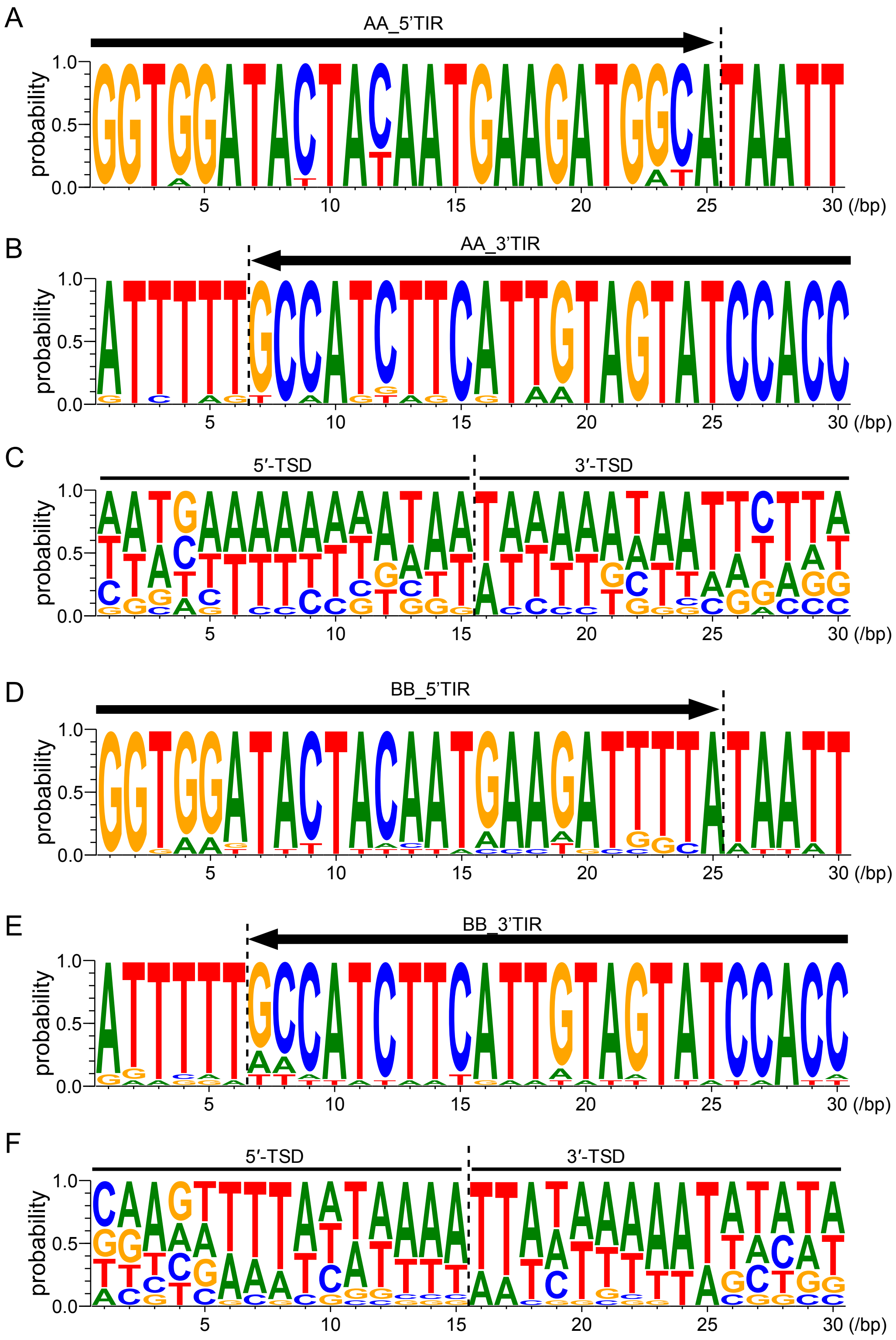

Supplementary Fig. 2 Conserved sequences of target site duplications (TSDs) and terminal inverted repeats (TIRs) in *AuMITE1* and *ApMITE1* elements. (A) and (B) 5' and 3' TIRs of *AuMITE1* elements. (C) Conserved sequences of TSDs within the flanking regions of *AuMITE1* members. (D) and (E) 5' and 3' TIRs of *ApMITE1* elements. (F) Conserved sequences of TSDs within the flanking regions of *ApMITE1* members. Black arrows and lines above the letters indicated the TSDs. The size of each letter indicates the frequency of the corresponding nucleotide.
