# Supplementary material for: Identification and characterization of transposable element AhMITE1 in the genomes of cultivated and two wild peanuts
Source: BMC Genomics. 2022 Jul 11;23:500. doi: 10.1186/s12864-022-08732-0 (PMC9277781; doi:10.1186/s12864-022-08732-0)
Supplement: Supplementary file 3 — Additional file 3: Supplementary fig 3. [file 12864_2022_8732_MOESM3_ESM.pdf]

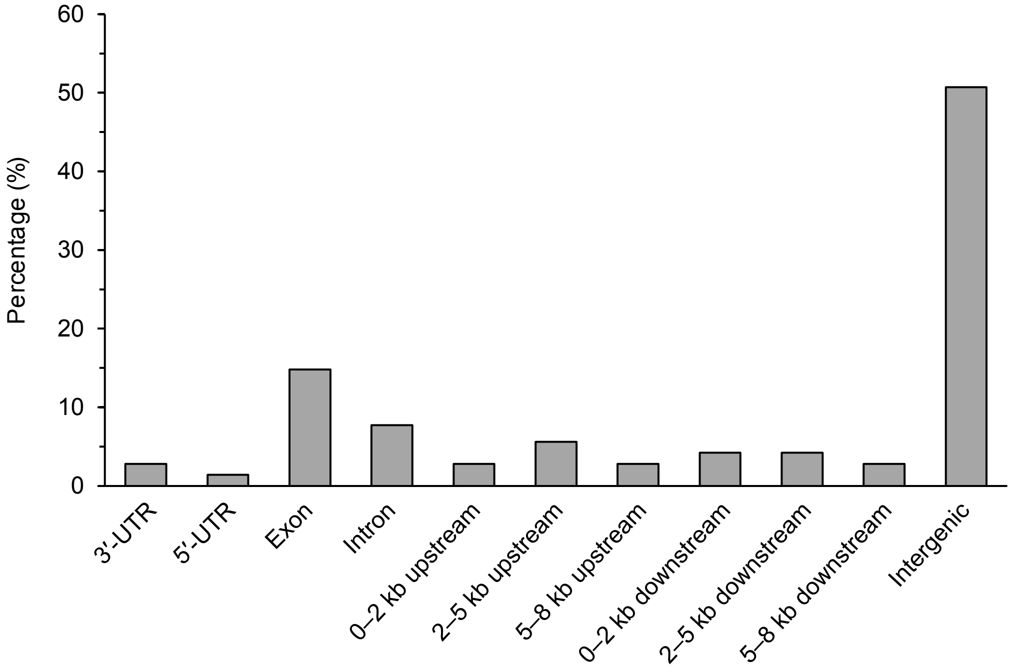

Supplementary Fig. 3 Insertion preferences of 142 randomly sequences in the genomes of *A. hypogaea* (cultivated peanut).
