# Supplementary material for: Identification and characterization of transposable element AhMITE1 in the genomes of cultivated and two wild peanuts
Source: BMC Genomics. 2022 Jul 11;23:500. doi: 10.1186/s12864-022-08732-0 (PMC9277781; doi:10.1186/s12864-022-08732-0)
Supplement: Supplementary file 4 — Additional file 4: Supplementary fig 4. [file 12864_2022_8732_MOESM4_ESM.pdf]

```
                     10        20        30        40        50        60        70        80        90       100       110       120
             ....|....|....|....|....|....|....|....|....|....|....|....|....|....|....|....|....|....|....|....|....|....|....|....|
AhMITE1_1    GGTGGATACTACAATGAAGATGGCATAATTGTCTTCATATGAGTATA----TTTCTTTTTGACCTTTGGATGATGGATTGTATGGTTAG---ATTTTGATATTTATAAAAGTGTTGTTTT
AhMITE1_2    GGTGGATACTACAATGAAGATGGCATAATTGTCTTCATATGAGTATA----TTTCTTTTTGACCTTTGGATGATGGATTGTATGGTTAG---ATTTTGATATTTATAAAAGTGTTGTTTT
AhMITE1_3    GGTGGATACTACAATGAAGATGGCATAATTGTCTTCATATGAGTATA----TTTCTTTTTGACCTTTGGATGATGGATTGTATGGTTAG---ATTTTGATATTTATAAATGTGTTGTTTT
AhMITE1_4    GGTGGATACTACAATGAAGATGGCATAATTGTCTTCATATGAGTATA----TTTCTTTTTGACCTTTGGATGATGGATTGTATGGTTAG---ATTTTGATATTTATAAATGTGTTGTTTT
AhMITE1_5    GGTGGATACTACAATGAAAATGGCATAATTATTTTCATATGAGTATA----TTTCTTTTTGACCTTTGGATGATGGATTGTATGGTTAG---ATTTTGATATTTATAAAAGTGTTGTTTT
AhMITE1_6    GGTGGATATTACAATGAAGATGGCATAATTGTCTTCATATGAGTATA----TTTCTTTTTGACTTTTAGATGATGGATTGTATGATTAG---ATTTTGATATTTATAAAAGTGTTGTTTT
AhMITE1_7    GGGGGATACTACAATGAAGATGTTATAATTGTCTTCATATGAATATA----TTGCTTTTTAACCTTTGGATGATGGATTGTATGGTTAG---ATTTTGAAATTTATAAAAGTGTTGTTTT
AhMITE1_8    AGTGGATACTACAATGAAGATTTTATAATTGTCTTCATGTGAATATA----TTTCTTTTTGACCCTTAAATGATGAATTGTATGGTTAG---ATTTTGATATTTATAAAAGTGTTGTTTT
AhMITE1_9    GGTGGATACTACAATAAAGATGGCATAATTGTCTTTATATGAGTATA-----TTTTTTTTGATTTTTGGATAATAAATTGTATAGTTAG---ATTTTGATATTTATAAAAGTGTTATTTT
AhMITE1_10   GGTGGATACTACAATGAAGATGGCATAATTGTCTTCATATGAGTATA----TTTCTTTTTGACCTTTGGATGATGGATTGTATGGTTAG---ATTTTGATATTTATAAAAGTGTTGTTTT
AhMITE1_11   GGTGGATACTACAATGAAGATGGCATAATTGTCTTCATATGAGTATA----TTTCTTTTTGACCTTTGGATGATGGATTGTATGGTTAG---ATTTTGATATTTATAAAAGTGTTGTTTT
AhMITE1_12   GGTGGATACTACAATGAAGATGGCATAATTGTCTTCATATGAGTATA----TTTCTTTTTGACCTTTGGATGATGGATTGTATGGTTAG---ATTTTGATATTTATAAAAGTGTTGTTTT
AhMITE1_13   GGTGGATACTACAATGAAGATGGCATAATTGTCTTCATATGAGTATA----TTTCTTTTTGACCTTTGGATGATGGATTGTATGGTTAG---ATTTTGATATTTATAAAAGTGTTGTTTT
AhMITE1_14   GGTGGATACTACAATGAAGATGGCATAATTGTCTTCATATGAGTATA----TTTCTTTTTGACCTTTGGATGATGGATTGTATGGTTAG---ATTTTGATATTTATAAATGTGTTGTTTT
AhMITE1_15   GGTGGATACTACAATGAAGATGGCATAATTGTCTTCATATGAGTATA----TTTCTTTTTGACCTTTGGATGATGGATTGTATGGTTAG---ATTTTGATATTTATAAATGTGTTGTTTT
AhMITE1_16   GGTGGATACTACAATGAAGATGGCATAATTATCTTCATATGAGTATA----TTTCTTTTTGACCTTTGGACGATGGAGTGTATGGTTAG---ATTTTGATATTTATAAAAGTGTTGTTTT
AhMITE1_17   GGGGGATACTACAATGAAGATGTTATAATTGTCTTCATATGAATATA----TTGCTTTTTAACCTTTGGATGATGGATTGTATGGTTAG---ATTTTGAAATTTATAAAAGTGTTGTTTT
AhMITE1_18   AGTGGATACTATAATAAAAATGGCATAATTGTCTTTATATGAATATA----TTTTTTTTTGACTTTTAGATGATGGATTGTATAGTTAG---ATTTTGATATTTATAAAAGTGTTGTTTT
AhMITE1_19   GGTGGATACTACAATGAAGATGGCATAATTGTCTTCATATGAGTATA----TTTCTTTTTGACCTTTGGATGATGGATTGTATGGTTAG---ATTTTGATATTTATAAAAGTGTTGTTTT
AhMITE1_20   GGTGGATACTACAATGAAGATGGCATAATTGTCTTCATATGAGTATA----TTTCTTTTTGACCTTTGGATGATGGATTGTATGGTTAG---ATTTTGATATTTATAAAAGTGTTGTTTT
AhMITE1_21   GGTGGATACTACAATGAAGATGGCATAATTGTCTTCATATGAGTATA----TTTCTTTTTGACCTTTGGATGATGGATTGTATGGTTAG---ATTTTGATATTTATAAAAGTGTTGTTTT
AhMITE1_22   GGTGGATACTACAATGAAGATGGCATAATTGTCTTCATATGAGTATA----TTTCTTTTTGACCTTTGGATGATGGATTGTATGGTTAG---ATTTTGATATTTATAAAAGTGTTGTTTT
AhMITE1_23   GGTGGATACTACAATGAAGATGGCATAATTGTCTTCATATGAGTATA----TTTCTTTTTGACCTTTGGATGATGGATTGTATGGTTAG---ATTTTGATATTTATAAAAGTGTTGTTTT
AhMITE1_24   GGTGGATACTACAATGAAGATGGCATAATTGTCTTCATATGAGTATA----TTTCTTTTTGACCTTTGGATGATGGATTGTATGGTTAG---ATTTTGATATTTATAAAAGTGTTGTTTT
AhMITE1_25   GGTGGATACTACAATGAAGATGGCATAATTGTCTTCATATGAGTATA----TTTCTTTTTGACCTTTGGATGATGGATTGTATGGTTAG---ATTTTGATATTTATAAAAGTGTTGTTTT
AhMITE1_26   GGTGGATACTACAATGAAGATGGCATAATTGTCTTCATATGAGTATA----TTTCTTTTTGACCTTTGGATGATGGATTGTATGGTTAG---ATTTTGATATTTATAAAAGTGTTGTTTT
AhMITE1_27   GGTGGATACTACAATGAAGATGGCATAATTGTCTTCATATGAGTATA----TTTCTTTTTGACCTTTGGATGATGGATTGTATGGTTAG---ATTTTGATATTTATAAAAGTGTTGTTTT
AhMITE1_28   AGTGGATACTACAATGAAGATGGCATAATTGTCTTCATATGAGTATA----TTTCTTTTTGACTTTTGGATGATGGATTGTATGGTTAG---ATTTTGATATTTATAAAAGTGTTGTTTT
AhMITE1_29   GGTGGATACTATAATGAAGATGGCATAATTGTCTTTATATGAGTATA----TTTCTTTTTTACCTTTGGATGATGGATTGTATGGTTAG---ATTTTGATATTTATAAAAGTGTTGTTTT
AhMITE1_30   GGTGGATACTACAATGAAGATGGCATAATTGTCTTCATATGAGTATA-----TTTTTTTTGATCTTTGGATGATGAATTGTATGGTTAG---ATTTTGATATTTATAAAAGTGTTGTTTT
AhMITE1_31   GGTGGATACTACAATAAAGATGGCATAATTGTCTTCATATGAGTATA----TTTCTTTTTGACCTTTGGATGATGGATTGTATGGTTAG---ATTTTGATATTTATAAAAGTGTTGTTTT
AhMITE1_32   GGTGGATACTACAATGAAGATGGCAAAAATATCTTCATATGAGTATA----TTTCTTTTTGACCTTTGGATGATGGATTGTATGGTTAG---ATTTTGATATTTATAAATGTGTTGTTTT
AhMITE1_33   GGTGGATACTACAATGAAGATGATATAATTGTCTTCATATGAGTATA----TTTTTTTTTGACCTTTGGATGATGAATTGTATGGTTAG---ATTTTGATATTTATAAAAATGTTGTTTT
AhMITE1_34   GGTGGATACTACAATGAAGATGGCATAATTGTCTTCATATGAGTATA----TTTCTTTTTGACCTTTGGATGATGGATTGTATGGTTAG---ATTTTGATATTTATAAAAGTGTTGTTTT
AhMITE1_35   GGTGGATACTACAATGAAGATGGCATAATTGTCTTCATATGAGTATA----TTTCTTTTTGACCTTTGGATGATGGATTGTATGGTTAG---ATTTTGATATTTATAAAAGTGTTGTTTT
AhMITE1_36   GGTGGATACTACAATGAAGATGGCATAATTGTCTTCATATGAGTATA----TTTCTTTTTGACCTTTGGATGATGGATTGTATGGTTAG---ATTTTGATATTTATAAAAGTGTTGTTTT
AhMITE1_37   GGTGGATACTACAATGAAGATGGCATAATTGTCTTCATATGAGTATA----TTTCTTTTTGACCTTTGGATGATGGATTGTATGGTTAG---ATTTTGATATTTATAAAAGTGTTGTTTT
AhMITE1_38   GGTGGATACTACAATGAAGATGGCATAATTGTCTTCATATGAGTATA----TTTCTTTTTGACCTTTGGATGATGGATTGTATGGTTAG---ATTTTGATATTTATAAATGTGTTGTTTT
AhMITE1_39   GGTGGATACTACAATGAAGATGGCAAAAATATCTTCATATGAGTATA----TTTCTTTTTGACCTTTGGATGATGGATTGTATGGTTAG---ATTTTGATATTTATAAAAGTGTTGTTTT
AhMITE1_40   GGTGGATACTACAATGAAGATGATATAATTGTCTTCATATGAGTATA----TTTTTTTTTGACCTTTGGATGATGGATTGTATGGTTAG---ATTTTGATATTTATAAAAATGTTGTTTT
AhMITE1_41   GGTGGATACTACAATGAAGATCTTATAATTGTCTTCATGTGAATATA----TTTCTTTTTGACCATTGGATGATGGATTGAATGGTTAG---ATTTTGATATTTATAAAAGTGTTATTTT
AhMITE1_42   GGTGGATACTACAATGAAGATCTTATAATTGTCTTCATGTGAATATA----TTTCTTTTTGACCATTGGATGATGGATTGAATGGTTAG---ATTTTGATATTTATAAAAGTGTTATTTT
AhMITE1_43   GGTGGATACTACAATGAAGATGGCATAATTGTCTTCATATGAGTATA----TTTCTTTTTGACCTTTGGATGATGGATTGTATGGTTAG---ATTTTGATATTTATAAAAGTGTTGTTTT
AhMITE1_44   GGTGGATACTACAATAAAGATGGCATAATTGTCTTCATATGAGTATA----TTTCTTTTTGACCTTTGGATGATGGATTGTATGGTTAG---ATTTTGATATTTATAAAAGTGTTGTTTT
AhMITE1_45   GGTGGATACTACAATAAAGATGGCATAATTGTCTTCATATGAGTATA----TTTCTTTTTGACCTTTGGATGATGGATTGTATGGTTAG---ATTTTGATATTTATAAAAGTGTTGTTTT
AhMITE1_46   GGTGGATACTACAATGAAGATGGCATAATTGTCTTCATATGAGTATA----TTTCTTTTTGACCTTTGGATGATGGATTGTATGGTTAG---ATTTTGATATTTATAAAAGTGTTGTTTT
AhMITE1_47   GGTGGATACTACAATGAAGATGGCATAATTGTCTTCATATGAGTATA----TTTCTTTTTGACCTTTGGATGATGGATTGTATGGTTAG---ATTTTGATAGTTATAAAAGTGTTGTTTT
AhMITE1_48   GGTGGATACTACAATGAAGATGGCATAATTGTCTTCATATGAGTATA----TTTCTTTTTGACCTTTGGATGATGGATTGTATGGTTAG---ATTTTGATATTTATAAATGTGTTGTTTT
AhMITE1_49   AGTGGATACTACAATGAAGATGGCATAATTGTCTTCATATGAGTATA----TTTCTTTTTGACCTTTGGATGATGGATTGTATGGTTAG---ATTTTGATATTTATAAAAGTGTTATTTT
AhMITE1_50   GGTGGATACTACAATGAAGATGGCATAATTGTCTTCATATGAGTATA----TTTCTTTTTGACCTTTGGATGATGGATTGTATGGTTAG---ATTTTGATATTTATAAAAGTGTTGTTTT
AhMITE1_51   GGTGGATACTACAATGAAGATGGCATAATTGTCTTCATATGAGTATA----TTTCTTTTTGACCTTTGGATGATGGATTGTATGGTTAG---ATTTTGATATTTATAAATGTGTTGTTTT
AhMITE1_52   GGTGGATACTACAATGAAGATGGCATAATTGTCTTCATATGAGTATA----TTTCTTTTTGACCTTTGGATGATGGATTGTATGGTTAG---ATTTTGATATTTATAAAAGTGTTGTTTT
AhMITE1_53   GGTGGATACTACAATGAAGATGGCATAATTGTCTTCATATGAGTATA----TTTCTTTTTGACCTTTGGATGATGGATTGTATGGTTAG---ATTTTGATATTTATAAAAGTGTTGTTTT
AhMITE1_54   GGTGGATACTACAATGAAGATGGCATAATTGTCTTCATATGAGTATA----TTTCTTTTTGACCTTTGGATGATGGATTGTATGGTTAG---ATTTTGATATTTATAAAAGTGTTGTTTT
AhMITE1_55   GGTGGATACTACAATGAAGATGGCATAATTGTCTTCATATGAGTATA----TTTCTTTTTGACCTTTGGATGATGGATTGTATGGTTAG---ATTTTGATATTTATAAAAGTGTTGTTTT
AhMITE1_56   GGTGGATACTACAATGAAGATGGCATAATTGTCTTCATATGAGTATA----TTTCTTTTTGACCTTTGGATGATGGATTGTATGGTTAG---ATTTTGATATTTATAAAAGTGTTGTTTT
AhMITE1_57   GGTGGATACTACAATGAAGATGATATAATTGTCTTCATATGAGTATA----TTTTTTTTTGACCTTTGGATGATGGATTGTATGGTTAG---ATTTTGATATTTATAAAAATGTTGTTTT
AhMITE1_58   GGTGGATACTACAATGAAGATCTTATAATTGTCTTCATGTGAATATA----TTTCTTTTTGACCATTGGATGATGGATTGAATGGTTAG---ATTTTGATATTTATAAAAGTGTTATTTT
AhMITE1_59   GGTGGATACTACAATGAAGATCTTATAATTGTCTTCATGTGAATATA----TTTCTTTTTGACCATTGGATGATGGATTGAATGGTTAG---ATTTTGATATTTATAAAAGTGTTATTTT
AhMITE1_60   GGTGGATACTACAATGAAGATCTTATAATTGTCTTCATGTGAATATA----TTTCTTTTTGACCATTGGATGATGGATTGAATGGTTAG---ATTTTGATATTTATAAAAGTGTTATTTT
AhMITE1_61   GGTGGATACTACAATGAAGATGGCATAATTGTCTTCATATGAGTATA----TTTCTTTTTGACCTTTGGATGATGGATTGTATGGTTAG---ATTTTGATATTTATAAAAGTGTTGTTTT
AhMITE1_62   GGTGGATACTACAATGAAGATGGCAAAAATATCTTCTCATGAAGATG----CTTTGGTTAAAAGTGTGGTTTAT---TTATTTAGCCAC---ACTTTAA-----ACAAAAACAACACTTT
AhMITE1_63   GGTGGATACTACAATGAAGATGGCATAATTGTCTTCATATGAGTATA----TTTCTTTTTGACCTTTGGATGATGGATTGTATGGTTAG---ATTTTGATATTTATAAAAGTGTTGTTTT
AhMITE1_64   GGTGGATACTACAATGAAGATGGCATAATTGTCTTCATATGAGTATA----TTTCTTTTTGACCTTTGGATGATGGATTGTATGGTTAG---ATTTTGATATTTATAAATGTGTTGTTTT
AhMITE1_65   GGTGGATACTATAATGAAGATGGCATAATTGTCTTCATATGAGTATA----TTTCTTTTTGACCTTTGGATGATGGATTGTATCGTTAG---ATTTTGATATTTATAAAAGTGTTGTTTT
AhMITE1_66   GGTGGATACTACAATGAAGATGGCATAATTGTCTTCATATGAGTATA----TTTCTTTTTGACCTTTGGATGATGGATTGTATGGTTAG---ATTTTGATATTTATAAATGTGTTGTTTT
AhMITE1_67   GGTGGATACTACAATGAAGATGGCATAATTGTCTTCATATGAGTATA----TTTCTTTTTGACCTTTGGATGATGGATTGTATGGTTAG---ATTTTGATATTTATAAATGTGTTGTTTT
AhMITE1_68   GGTGGATACTACAATGAAGATGGCATAATTATTTTCATATGAGTATA----TTTCTTTTTGACCTTTGGATGATGGATTGTATGGTTAG---ATTTTGATATTTATAAAAGTGTTGTTTT
AhMITE1_69   TGTGGATACTACAATGAAAATTTTATAATTGTCTTCATATGAATATA----TTTCTTTTTGACCCTTGGATGATGGATTGTATGGTTGA---ATTTTGATATTTATAAAAGTGTTATTTT
AhMITE1_70   GGTGGATACTACAATGAAGATGGCATAATTGTCTTCATATGAGTATA----TTTCTTTTTGACCTTTGGATGATGGATTGTATGGTTAG---ATTTTGATATTTATAAATGTGTTGTTTT
AhMITE1_71   GGTGGATACTACAATGAAGATGATATAATTGTCTTCATATGAGTATA----TTTTTTTTTGACCTTTGGATGATGGATTGTATGGTTAG---ATTTTGATATTTATAAAAATGTTGTTTT
AhMITE1_72   GGTGGATACTACAATGAAGATGGCATAATTGTCTTCATATGAGTATA-----TTTTTTTTGACCTTTAGACGATGGATTGTATGGTTAG---ATTTTGATATTTATAAAAGTGTTGTTTT
AhMITE1_73   GGTGGATACTACAATGAAGATGGCATAATTGTCTTCATATGAGTATA----TTTCTTTTTGACCTTTGGATGATGGATTGTATGGTTAG---ATTTTGATATTTATAAAAGTGTTGTTTT
AhMITE1_74   GGTGGATACTACAATGAAGATGGCATAATTGTCTTCATATGAGTATA----TTTCTTTTTGACCTTTGGATGATGGATTGTATGGTTAG---ATTTTGATATTTATAAATGTGTTGTTTT
AhMITE1_75   GGTGGATACTACAATGAAGATGGCATAATTGTCTTCATATGAGTATA----TTTCTTTTTGACCTTTGGATGATGGATTGTATGGTTAG---ATTTTGATATTTATAAATGTGTTGTTTT
AhMITE1_76   GGTGGATACTACAATGAAGATCTTATAATTGTCTTCATGTGAATATA----TTTCTTTTTGACCATTGGATGATGGATTGAATGGTTAG---ATTTTGATATTTATAAAAGTGTTATTTT
AhMITE1_77   GGTGGATACTACCATGAAGATTTTATAATTGTCTTCATGTAAATATA----TTTCTTTTTGATCCTTGGATGATGGATTGTATGGTTAG---ATTTTGATATTTATAAAAGTGTTGTTTT
AhMITE1_78   GGTGGATACTACAATGAAGATTTTATAATTGTCTTCATATGAATATATTATTTATTTTTTGATCCTTGGATGATGGATTGTATGGTTAG---ATTTTGATATTTATAAAAGTGTTGTTTT
AhMITE1_79   GGTGGATACTACAATGAAGATTTTATAATTGTCTTCATGTGAATATA----TTTCTTTTTGACCATTGAATGATGGATTGTATGGTTAG---ATTTTGATATTTATAAAAGTGTTATTTT
AhMITE1_80   AGTGGATATTACAATGAAGATTTTATAATTATCTTCATGTGAATATA----TTTCTTTTTGACCCTTGGATGATAGATTGTATGGTTAG---ATTTTGATATTTATAAAAGTGTTGTTTT
AhMITE1_81   GGTGGATACTACAATGAAGATTTTATAATTGTCTTCATGTGAATATA----TTTCTTTTTGACTCTTGGATGATGAATTATATAGTTAA---ATTTTGATATTTATAAAATTGTTGTTTT
AhMITE1_82   GGTGGATACTACAATGAAGATGGCATAATTATTTTCATATGAGTATA----TTTCTTTTTGACCTTTGGATGATGGATTGTATGGTTAG---ATTTTGATATTTATAAAAGTGTTGTTTT
AhMITE1_83   GGTGGATACTACAATGAAGATTTTATAATTGTCTTTATGTGAATATA----TTTCTTTTTGACCTTTGGATGATGGATTGTATGGTTAG---ATTTTGATATTTATAAAAGTGTTATTTT
AhMITE1_84   GGTGGATACTACAATGAAGATGGCATAATTGTCTTCATATGAGTATA----TTTCTTTTTGACCTTTGGATGATGGATTGTATGGTTAG---ATTTTGATATTTATAAAAGTGTTGTTTT
AhMITE1_85   GGTGGATACTACAATGAAGATGGCATAATTGTCTTCATATGAGTATA----TTTCTTTTTGACCTTTGGATGATGGATTGTATGGTTAG---ATTTTGATATTTATAAAAGTGTTGTTTT
AhMITE1_86   GGTGGATACTACAATGAAGATGGCATAATTGTCTTCATATGAGTATA----TTTCTTTTTGACCTTTGGATGATGGATTGTATGGTTAG---ATTTTGATATTTATAAAAGTGTTGTTTT
AhMITE1_87   GGTGGATACTACAATGAAGATGGCATAATTGTCTTCATATGAGTATA----TTTTTTTTTGACCTTTGGATGATGGATTGTATGGTTAG---ATTTTGATATTTATAAAAGTGTTGTTTT
AhMITE1_88   GGTGGATACTACAATGAAGATGGCATAATTGTCTTCATATGAGTATA----TTTCTTTTTGACCTTTGGATGATGGATTGTATGGTTAG---ATTTTGATATTTATAAATGTGTTGTTTT
AhMITE1_89   GGTGGATACTACAATGAAGATGGCATAATTGTCTTCATATGAGTATA----TTTCTTTTTGACCTTTGGATGATGGATTGTATGGTTAG---ATTTTGATATTTATAAATGTGTTGTTTT
AhMITE1_90   AGTGGATACTACAATGAAGATTTTATAATTGTCTTCATGTGAATATA----TTTCTTTTTGACCCTTAAATGATGAATTGTATGGTTAG---ATTTTGATATTTATAAAAGTGTTGTTTT
```

```
              10        20        30        40        50        60        70        80        90       100       110       120
     ....|....|....|....|....|....|....|....|....|....|....|....|....|....|....|....|....|....|....|....|....|....|....|....|
AhMITE1_91   GGTGGATACTACAATAAAGATTTTATAATTGTCTTCATGTGAATATA----TTTCTTTTTGACTCTTCGATGATGGATTGTATGGTTAG---ATTTTGATATTTATAAAAGTGTTGTTTT
AhMITE1_92   GGTGGATACTACCATGAAGATTTTATAATTATCTTCGTGTGAATATA----TTTCTTTTTGACCCTTGGATGATGGATTGTATGGTTAG---ATTTTGATATTTATAAAAGTG---TTTT
AhMITE1_93   GGTGAATACTACAATGAAAATTTTATAATTGTCTTAATGTGAATATA---TTTTTTTTTTTACCCTTGGATGATGGATTGTATGTTTAG---ATTTTGATATTTATAAAATTGTTATTTT
AhMITE1_94   GGTGGATACTACAATGAAGATGGCATAATTGTCTTCATATGAGTATA----TTTCTTTTTGACCTTTGGATGATGGATTGTATGGTTAG---ATTTTGATATTTATAAAAGTGTTGTTTT
AhMITE1_95   GGTGGATACTACAATGAAGATGGCATAATTGTCTTCATATGAGTATA----TTTCTTTTTGACCTTTGGATGATGGATTGTATGGTTAG---ATTTTGATATTTATAAAAGTGTTGTTTT
AhMITE1_96   GGTGGATACTACAATGAAGATGGCAAAAATATCTTCTCATGAAGATG----CTTTGGTTAAAAGTGTGGTTTAT---TTATTTAGCCAC---ACTTTAA-----ACAAAAACAACACTTT
AhMITE1_97   GGTGGATACTACAATAAAGATGGCATAATTGTCTTCATATGAGTATA----TTTCTTTTTGACCTTTGGATGATGGATTGTATGGTTAG---ATTTTGATATTTATAAAAGTGTTGTTTT
AhMITE1_98   GGTGGATACTACAATGAAGATGGCATAATTGTCTTCATATGAGTATA----TTTCTTTTTGACCTTTGGATGATGGATTGTATGGTTAG---ATTTTGATATTTATAAAAGTGTTGTTTT
AhMITE1_99   GGTGGATACTACAATGAAGATGGCATAATTGTCTTCATATGAGTATA----TTTCTTTTTGACCTTTGGATGATGGATTGTATGGTTAG---ATTTTGATATTTATAAAAGTGTTGTTTT
AhMITE1_100  GGTGGATACTACAATGAAGATGGCATAATTGTCTTCATATGAGTATA----TTTCTTTTTGACCTTTGGATGATGGATTGTATGGTTAG---ATTTTGATATTTATAAAAGTGTTGTTTT
AhMITE1_101  GGTGGATACTACAATGAAGATGGCATAATTGTCTTCATATGAGTATA----TTTCTTTTTGACCTTTGGATGATGGATTGTATGGTTAG---ATTTTGATATTTATAAAAGTGTTGTTTT
AhMITE1_102  GGTGGATACTACAATGAAGATGGCATAATTGTCTTCATATGAGTATA----TTTCTTTTTGACCTTTGGATGATGGATTGTATGGTTAG---ATTTTGATATTTATAAAAGTGTTGTTTT
AhMITE1_103  GGTGGATACTACAATGAAGATGGCATAATTGTCTTCATATGAGTATA----TTTCTTTTTGACCTTTGGATGATGGATTGTATGGTTAG---ATTTTGATATTTATAAAAGTGTTGTTTT
AhMITE1_104  GGTAGATACTACAATGAAGATTTTATAATTGTCTTCATGTGAATATA----TTTCTTTTTGACCCTTGGATGATGGATTGTATGGTTAG---ATTTTGATATTTATAAAAGTGTTATTTT
AhMITE1_105  GGTGGATACTACAATGAAGATTTTATAATTGTCTTCGTATGAATATA----TTGCTTTTTGATCCTTGGATGATGGATTGTATGGTTAG---ATTTTGATATTTATAAAAATGTTATTTT
AhMITE1_106  GGTAGATACTACAATGAAGATTTTATAATTGTCTTCATGTGAATATA----TTTCTTTTTTATCCTTGGATGCTAGATTGTATGATTAG---ATTTTGATATTTATAAAAGTGTTGTTTT
AhMITE1_107  GGTGGATACTACAATGAAGATGGCATAATTGTCTTCATATGAGTATA----TTTCTTTTTGACCTTTGGATGATGGATTGTATGGTTAG---ATTTTGATATTTATAAAAGTGTTGTTTT
AhMITE1_108  GGTGGATACTACAATGAAGATGGCATAATTGTCTTCATATGAGTATA----TTTCTTTTTGACCTTTGGATGATGGATTGTATGGTTAG---ATTTTGATATTTATAAAAGTGTTGTTTT
AhMITE1_109  GGTGGATACTACAATGAAGATGGCATAATTGTCTTCATATGAGTATA----TTTCTTTTTGACCTTTGGATGATGGATTGTATGGTTAG---ATTTTGATATTTATAAAAGTGTTGTTTT
AhMITE1_110  GGTGGATACTACAATGAAGATGGCATAATTGTCTTCATATGAGTATA----TTTCTTTTTGACCTTTGGATGATGGATTGTATGGTTAG---ATTTTGATATTTATAAAAGTGTTGTTTT
AhMITE1_111  GGTGGATACTACAATGAAGATGGCATAATTGTCTTCATATGAGTATA----TTTCTTTTTGACCTTTGGATGATGGATTGTATGGTTAG---ATTTTGATATTTATAAATGTGTTGTTTT
AhMITE1_112  GGTGGATACTACAATGAAGATGGCAAAAATATCTTCATATGAGTATA----TTTCTTTTTGACCTTTGGATGATGGATTGTATGGTTAG---ATTTTGATATTTATAAAAGTGTTGTTTT
AhMITE1_113  GGTGGATACTACAATCAAGATTTTATAATTGTTTTTATATGAATATA----TTTCTTTTTGACCTTTGGATGATGGATTGTATGATTAG---ATTTTGATATTTATAAAAGTGTTGTTTT
AhMITE1_114  GGTGGATACTACAATAAAGATTTTATAATTGTCTTTATATGAATATA----TTTCTTTTTGACTCTTGGATGATAAATTGTATGGTTAG---ATTTTGATATTTATAAAAGTGTTGTTTT
AhMITE1_115  GGTGGATACTACAATGAAGATGGCATAATTGTCTTCATATGAGTATA----TTTCTTTTTGACCTTTGGATGATGGATTGTATGGTTAG---ATTTTGATATTTATAAAAGTGTTGTTTT
AhMITE1_116  GGTGGATACTACAATGAAGATGGCATAATTGTCTTCATATGAGTATA----TTTCTTTTTGACCTTTGGATGATGGATTGTATGGTTAG---ATTTTGATATTTATAAAAGTGTTGTTTT
AhMITE1_117  GGTGGATACTACAATGAAGATGGCATAATTGTCTTCATATGAGTATA----TTTCTTTTTGACCTTTGGATGATGGATTGTATGGTTAG---ATTTTGATATTTATAAAAGTGTTGTTTT
AhMITE1_118  GGTGGATACTACAATGAAGATGGCATAATTGTCTTCATATGAGTATA----TTTCTTTTTGACCTTTGGATGATGGATTGTATGGTTAG---ATTTTGATATTTATAAAAGTGTTGTTTT
AhMITE1_119  GGGGGATACTACAATGAAGATGTTATAATTGTCTTCATATGAATATA----TTGCTTTTTAACCTTTGGATGATGGATTGTATGGTTAG---ATTTTGAAATTTATAAAAGTGTTGTTTT
AhMITE1_120  GGGGGATACTACAATGAAGATGTTATAATTGTCTTCATATGAATATA----TTGCTTTTTAACCCTTGGATGATGGATTGTATGGTTAG---ATTTTGAAATTTATAAAAGTGTTGTTTT
AhMITE1_121  GGTGGATACTACAATGAATATTTTATAATTGTTTTCATGTGAATATA----TTTCTTTTTGACCCTTGGATGATGGATTGTATGGTTAG---ATTTTGATATTTATAAAAGTGTTGTTTT
AhMITE1_122  GGTGGATACTACAATGAAGATCTTATAATTGTCTTCATGTGAATATA----TTTCTTTTTGACCATTGGATGATGGATTGAATGGTTAGATTATTTTGATATTTATAAAAGTGTTATTTT
AhMITE1_123  GGTGGATACTACAATGAAGATGGTATAATTGTCTTTATGTGAATATA----TTTTTTTTTTACCCTTAGATGATAGATTGTATGGTTAG---ATTTTGATATTTATAGAAGTGTTATTTT
AhMITE1_124  GGTAGATACTACAATGAAGATTTTATAATTGTCTTCATGTGAATATA----TTTCTTTTTGACTCTTGGATGATGAATTGTATGATTAG---ATTTTGATATTTATAAAAGTGTTATTTT
AhMITE1_125  GGTGGATACTACAATGAAGATGGCATAATTGTCTTCATATGAGTATA----TTTCTTTTTGACCTTTGGATGATGGATTGTATGGTTAG---ATTTTGATATTTATAAAAGTGTTGTTTT
AhMITE1_126  TATGGATACTACAATGAAGATGGCATAATTGTCTTCATATGAGTATA----TTTCTTTTTGACCTTTGGATGATGGATTGTATGGTTAG---ATTTTGATATTTATAAAAGTGTTGTTTT
AhMITE1_127  GGTGGATACTACAATGAAGATGGCATAATTGTCTTCATATGAGTATA----TTTCTTTTTGACCTTTGGATGATGGATTGTATGGTTAG---ATTTTGATATTTATAAATGTGTTGTTTT
AhMITE1_128  GGTGGATACTACAATGAAGATGGCATAATTATTTTCATATGAGTATA----TTTCTTTTTGACCTTTGGATGATGGATTGTATGGTTAG---ATTTTGATATTTATAAAAGTGTTGTTTT
AhMITE1_129  GGTGGATACTACAATGAAGATGGCATAATTATTTTCATATGAGTATA----TTTCTTTTTGACCTTTGGATGATGGA-TGTATGGTTAG---ATTTTGATATTTATAAAAGTGTTGTTTT
AhMITE1_130  GGTGGATACTACAATGAAGATTTTATAATTGTCTTCATGTGAATATA-----TTTTTTTTTACCCTTGGATGATGGATTGTATGGTTAG---ATTTTGATATTTATAAAAGTGTTGTTTT
AhMITE1_131  GGTGGATACTACAATGAAGATGGCATAATTGTCTTCATATGAGTATA----TTTCTTTTTGACCTTTGGATGATGGATTGTATGGTTAG---ATTTTGATATTTATAAAAGTGTTGTTTT
AhMITE1_132  GGTGGATACTACAATGAAGATGGCATAATTGTCTTCATATGAGTATA----TTTCTTTTTGACCTTTGGATGATGGATTGTATGGTTAG---ATTTTGATATTTATAAAAGTGTTGTTTT
AhMITE1_133  GGTGGATACTACAATGAAGATGGCATAATTGTCTTCATATGAGTATA----TTTCTTTTTGACCTTTGGATGATGGATTGTATGGTTAG---ATTTTGATATTTATAAAAGTGTTGTTTT
AhMITE1_134  GGTGGATACTACAATGAAGATGGCATAATTGTCTTCATATGAGTATA----TTTCTTTTTGACCTTTGGATGATGGATTGTATGGTTAG---ATTTTGATATTTATAAAAGTGTTGTTTT
AhMITE1_135  GGTGGATATTACAATGAAGATGGCATAATTGTCTTCATATGAGTATA----TTTCTTTTTGACCTTTGGATGATGGATTGTATGGTTAG---ATTTTGATATTTATAAAAGTGTTGTTTT
AhMITE1_136  GGTGGATACTACAATGAAGATGGCATAATTGTCTTCATATGAGTATA----TTTCTTTTTGACCTTTGGATGATGGATTGTATGGTTAG---ATTTTGATATTTATAAATGTGTTGTTTT
AhMITE1_137  GGTGGATACTACAATGAAGGTGGCATAATTGTCTTCATATGAGTATA----TTTCTTTTTGACCTTTGGATGATGGATTGTATGGTTAG---ATTTTGATATTTATAAATGTGTTGTTTT
AhMITE1_138  GGTGGATACTACAATGAAGATTTTATAATTGTCTTTATGTGAATATA---TTTTTTTTTTGACCTTTGGATAATGGATTGTATGGTTAC---ATTTTGATATTTATAAAAGTGTTGTTTT
AhMITE1_139  GGTGGATACTACAATGAAGATGGCATAATTGTCTTCATATGAGTATA----TTTCTTTTTGACCTTTGGATGATGGATTGTATGGTTAG---ATTTTGATATTTATAAAAGTGTTGTTTT
AhMITE1_140  GGTGGATACTACAATGAAGATGGCATAATTGTCTTCATATGAGTATA----TTTCTTTTTGACCTTTGGATGATGGATTGTATGGTTAG---ATTTTGATATTTATAAAAGTGTTGTTTT
AhMITE1_141  GGTGGATACTACAATGAAGATGGCATAATTGTCTTCATATGAGTATA----TTTCTTTTTGACCTTTGGATGATGGATTGTATGGTTAG---ATTTTGATATTTATAAAAGTGTTGTTTT
AhMITE1_142  GGTGGATACTACAATGAAGATTTTATAATTGTTTTCATATGAATATA----TTATTTTTTGACCCTTGGATGATAGATTGTATGGTTAG---ATTTTGATATTCATAAAAGTGTTGTTTT

                 130       140       150       160       170       180       190       200       210       220
     ....|....|....|....|....|....|....|....|....|....|....|....|....|....|....|....|....|....|....|....|.
AhMITE1_1    TGT-----TTAAAGTGTGGCTAAATAA---ATAAACCACACTTTTAACCAAAGCATCTTCATGAGAAGATATTTTTGCCATCTTCA-TTGTAGTATCCACC
AhMITE1_2    TGT-----TTAAAGTGTGGCTAAATAA---ATAAACCACACTTTTAACCAAAGCATCTTCATGAGAAGATATTTTTGCCATCTTCA-TTGTAGTATCCACC
AhMITE1_3    TGT-----TTAAAGTGTGGCTAAATAA---ATAAACCACACTTTTAACTAAAGCATCTTCATGAGAAGATATTTTTGCCATCTTCA-TTGTAGTATCCACC
AhMITE1_4    TGT-----TTAAAGTGTGGCTAAATAA---ATAAACCACACTTTTAACTAAAGCATCTTCATGAGAAGATATTTTTGCCATCTTCA-TTGTAGTATCCACC
AhMITE1_5    TGT-----TTAAAGTGTGGCTAAATAA---ATAAACCACACTTTTAACCAAAGCATCTTCATAAGAAGATATTTTTGCCATCTTCA-TAGTAGTATCCACC
AhMITE1_6    TGT-----TTAAAGTGTGGTTAAATAA---ATAAACCACACTTTTAACTAAAGCTTCTTCATGAGAAGATATTTTTGCCATCTTCA-TTATAGTATCCACC
AhMITE1_7    TGT-----TTAAAGTGTGGCTAAATAA---ATAAACTACACTTTTAACCAAAGCATCTTCATGAGAAGATATTTTTGCCATCTTCA-TTGTAGTATCCACC
AhMITE1_8    TGT-----TTAAAGTGTGGCTAAATAA---ATAAACCACACTTTTAACCAAAGCATCTTCATGAGAAGATATTTTTGCCATCTTCA-TTGTAGTATCCACC
AhMITE1_9    TAT-----TTAAAGTGTGGCTAAATAA---ATAAATCACACTTTTAATTAAAGCATCTTTATAAAAAGATATTTTTGCCATCTTTATTTTTAGTATCCACC
AhMITE1_10   TGT-----TTAAAGTGTGGCTAAATAA---ATAAACCACACTTTTAACCAAAGCATCTTCATGAGAAGATATTTTTGCCATCTTCA-TTGTAGTATCCACC
AhMITE1_11   TGT-----TTAAAGTGTGGCTAAATAA---ATAAACCACACTTTTAACCAAAGCATCTTCATGAGAAGATATTTTTGCCATCTTCA-TTGTAGTATCCACC
AhMITE1_12   TGT-----TTAAAGTGTGGCTAAATAA---ATAAACCACACTTTTAACCAAAGCATCTTCATGAGAAGATATTTTTGCCATCTTCA-TTGTAGTATCCACC
AhMITE1_13   TGT-----TTAAAGTGTGGCTAAATAA---ATAAACCACACTTTTAACCAAAGCATCTTCATGAGAAGATATTTTTGCCATCTTCA-TTGTAGTATCCACC
AhMITE1_14   TGT-----TTAAAGTGTGGCTAAATAA---ATAAACCACACTTTTAACTAAAGCATCTTCATGAGAAGATATTTTTGCCATCTTCA-TTGTAGTATCCACC
AhMITE1_15   TGT-----TTAAAGTGTGGCTAAATAA---ATAAACCACACTTTTAACTAAAGCATCTTCATGAGAAGATATTTTTGCCATCTTCA-TTGTAGTATCCACC
AhMITE1_16   TGT-----TTAAAGTGTGGCTAAATAA---ATAAACCACACTTTTAACCAAAGCATCTTCATGAGAAGATATTTTTGCCATCTTCG-TTGTAGTATCCACC
AhMITE1_17   TGT-----TTAAAGTGTGGCTAAATAA---ATAAACTACACTTTTAACCAAAGCATCTTCATGAGAAGATATTTTTGCCATCTTCA-TTGTAGTATCCACC
AhMITE1_18   TGT-----TTAAAATATGGCTAAATAA---ATAAACCACACTTTTAACCAAAGCATCTTCATGAGAAGATAATTTTGCCATCTTCA-TTGTAGTATCCACC
AhMITE1_19   TGT-----TTAAAGTGTGGCTAAATAA---ATAAACCACACTTTTAACCAAAGCATCTTCATGAGAAGATATTTTTGCCATCTTCA-TTGTAGTATCCACC
AhMITE1_20   TGT-----TTAAAGTGTGGCTAAATAA---ATAAACCACACTTTTAACCAAAGCATCTTCATGAGAAGATATTTTTGCCATCTTCA-TTGTAGTATCCACC
AhMITE1_21   TGT-----TTAAAGTGTGGCTAAATAA---ATAAACCACACTTTTAACCAAAGCATCTTCATGAGAAGATATTTTTGCCATCTTCA-TTGTAGTATCCACC
AhMITE1_22   TGT-----TTAAAGTGTGGCTAAATAA---ATAAACCACACTTTTAACCAAAGCATCTTCATGAGAAGATATTTTTGCCATCTTCA-TTGTAGTATCCACC
AhMITE1_23   TGT-----TTAAAGTGTGGCTAAATAA---ATAAACCACACTTTTAACCAAAGCATCTTCATGAGAAGATATTTTTGCCATCTTCA-TTGTAGTATCCACC
AhMITE1_24   TGT-----TTAAAGTGTGGCTAAATAA---ATAAACCACACTTTTAACCAAAGCATCTTCATGAGAAGATATTTTTGCCATCTTCA-TTGTAGTATCCACC
AhMITE1_25   TGT-----TTAAAGTGTGGCTAAATAA---ATAAACCACACTTTTAACCAAAGCATCTTCATGAGAAGATATTTTTGCCATCTTCA-TTGTAGTATCCACC
AhMITE1_26   TGT-----TTAAAGTGTGGCTAAATAA---ATAAACCACACTTTTAACCAAAGCATCTTCATGAGAAGATATTTTTGCCATCTTCA-TTGTAGTATCCACC
AhMITE1_27   TGT-----TTAAAGTGTGGCTAAATAA---ATAAACCACACTTTTAACCAAAGCATCTTCATGAGAAGATA-TTTTGCCATCTTCA-TTGTAGTATCCACC
AhMITE1_28   TGT-----TTAAAGTGTGGCTAAATAA---ATAAACCACACTTTTAACCAAAGCATCTTCATGAGAAGATATTTTTGCCATCTTCA-TTGTAATATCCACC
AhMITE1_29   TGT-----TTAAAGTGTGGCTAAATAA---ATAAACCACACTTTTAACCAAAGCATCTTCATGAGAAGATATTTTTGCCATCTTCA-TTATAGTATCCACC
AhMITE1_30   TGT-----TTAAAGTGTGGCTAAATAA---ATAAACCACACTTTTAACCAAAACATCTTCATGAGAAGATATTTTTGCCATCTTCA-TTGTAGTATCCACC
AhMITE1_31   TGT-----TTAAAGTGTGGCTAAATAA---ATAAACCACACTTTTAACCAAAGCATCTTCATGAGAAGATATTTTTGCCATCTTCA-TTGTAGTATCCACC
AhMITE1_32   TGT-----TTAAAGTGTGGCTAAATAA---ATAAACCACACTTTTAACTAAAGCATCTTCATGAGAAGATATTTTTGCCATCTTCA-TTGTAGTATCCACC
AhMITE1_33   TGT-----TTAAAGTGTGGCTAAATAA---ATAAACTACACTTTTAACCAAAGCATCTTCATAAGAAGATATTTTTGCCATCTTCA-TTGTAGTATCCACC
AhMITE1_34   TGT-----TTAAAGTGTGGCTAAATAA---ATAAACCACACTTTTAACCAAAGCATCTTCATGAGAAGATATTTTTGCCATCTTCA-TTGTAGTATCCACC
AhMITE1_35   TGT-----TTAAAGTGTGGCTAAATAA---ATAAACCACACTTTTAACCAAAGCATCTTCATGAGAAGATATTTTTGCCATCTTCA-TTGTAGTATCCACC
AhMITE1_36   TGT-----TTAAAGTGTGGCTAAATAA---ATAAACCACACTTTTAACCAAAGCATCTTCATGAGAAGATATTTTTGCCATCTTCA-TTGTAGTATCCACC
AhMITE1_37   TGT-----TTAAAGTGTGGCTAAATAA---ATAAACCACACTTTTAACCAAAGCATCTTCATGAGAAGATATTTTTGCCATCTTCA-TTGTAGTATCCACC
```

```
                     130       140       150       160       170       180       190       200       210       220
             ....|....|....|....|....|....|....|....|....|....|....|....|....|....|....|....|....|....|....|....|.
AhMITE1_38   TGT-----TTAAAGTGTGGCTAAATAA---ATAAACCACACTTTTAACTAAAGCATCTTCATGAGAAGATATTTTTGCCATCTTCA-TTGTAGTATCCACC
AhMITE1_39   TGT-----TTAAAGTGTGGCTAAATAA---ATAAACCACACTTTTAACCAAAGCATCTTCATGAGAAGATATTTTTGCCATCTTCA-TTGTAGTATCCACC
AhMITE1_40   TGT-----TTAAAGTGTGGCTAAATAA---ATAAACTACACTTTTAACCAAAGCATCTTCATAAGAAGATATTTTTGCCATCTTCA-TTGTAGTATCCACC
AhMITE1_41   TGT-----TTAAAGTGTGGCTAAATAA---ATAAACCACACTTTTAACCAAAGCATCTTCATGAGAAGATATTTTTGCCATCTTCA-TTGTAGTATCCACC
AhMITE1_42   TGT-----TTAAAGTGTGGCTAAATAA---ATAAACCACACTTTTAACCAAAGCATCTTCATGAGAAGATATTTTTGCCATCTTCA-TTGTAGTATCCACC
AhMITE1_43   TGT-----TTAAAGTGTGGCTAAATAA---ATAAACCACACTTTTAACCAAAGCATCTTCATGAGAAGATATTTTTGCCATCTTCA-TTGTAGTATCCACC
AhMITE1_44   TGT-----TTAAAGTGTGGCTAAATAA---ATAAACCACACTTTTAACCAAAGCATCTTCATGAGAAGATATTTTTGCCATCTTCA-TTGTAGTATCCACC
AhMITE1_45   TGT-----TTAAAGTGTGGCTAAATAA---ATAAACCACACTTTTAACCAAAGCATCTTCATGAGAAGATATTTTTGCCATCTTCA-TTGTAGTATCCACC
AhMITE1_46   TGT-----TTAAAGTGTGGCTAAATAA---ATAAACCACACTTTTAACCAAAACATCTTCATGAGAAGATATTTTTGCCATCTTCA-TTGTAGTATCCACC
AhMITE1_47   TGT-----TTAAAGTGTGGCTAAATAA---ATAAACCACACTTTTAACCAAAGCATCTTCATGAGAAGATATTTTTGCCATTTTCA-TTGTAGTATCCACC
AhMITE1_48   TGT-----TTAAAGTGTGGCTAAATAA---ATAAACCACACTTTTAACTAAAGCATCTTCATGAGAAGATATTTTTGCCATCTTCA-TTGTAGTATCCACC
AhMITE1_49   TGT-----TTAAAGTGTGGCTAAATAA---ATAAACCACACTTTTAACCAAAACATCTTCATGAAAAAATATTTTTGCCATCTTCA-TAGTAGTATCCACC
AhMITE1_50   TGT-----TTAAAGTGTGGCTAAATAA---ATAAACCACACTTTTAACCAAAGCATCTTCATGAGAAGATATTTTTGCCATCTTCA-TTGTAGTATCCACC
AhMITE1_51   TGT-----TTAAAGTGTGGCTAAATAA---ATAAACCACACTTTTAACTAAAGCATCTTCATGAGAAGATATTTTTGCCATCTTCA-TTGTAGTATCCACC
AhMITE1_52   TGT-----TTAAAGTGTGGCTAAATAA---ATAAACCACACTTTTAACTAAAGTATCTTCATGAGAAGATATTTTTGCCATCTTCA-TTGTAGTATCCACC
AhMITE1_53   TGT-----TTAAAGTGTGGCTAAATAA---ATAAACCACACTTTTAACCAAAGCATCTTCATGAGAAGATATTTTTGCCATCTTCA-TTGTAGTATCCACC
AhMITE1_54   TGT-----TTAAAGTGTGGCTAAATAA---ATAAACCACACTTTTAACCAAAGCATCTTCATGAGAAGATATTTTTGCCATCTTCA-TTGTAGTATCCACC
AhMITE1_55   TGT-----TTAAAGTGTGGCTAAATAA---ATAAACCACACTTTTAACCAAAGCATCTTCATGAGAAGATATTTTTGCCATCTTCA-TTGTAGTATCCACC
AhMITE1_56   TGT-----TTAAAGTGTGGCTAAATAA---ATAAACCACACTTTTAACTAAAGTATCTTCATGAGAAGATATTTTTGCCATCTTCA-TTGTAGTATCCACC
AhMITE1_57   TGT-----TTAAAGTGTGGCTAAATAA---ATAAACTACACTTTTAACCAAAGCATCTTCATAAGAAGATATTTTTGCCATCTTCA-TTGTAGTATCCACC
AhMITE1_58   TGT-----TTAAAGTGTGGCTAAATAA---ATAAACCACACTTTTAACCAAAGCATCTTCATGAGAAGATATTTTTGCCATCTTCA-TTGTAGTATCCACC
AhMITE1_59   TGT-----TTAAAGTGTGGCTAAATAA---ATAAACCACACTTTTAACCAAAGCATCTTCATGAGAAGATATTTTTGCCATCTTCA-TTGTAGTATCCACC
AhMITE1_60   TGT-----TTAAAGTGTGGCTAAATAA---ATAAACCACACTTTTAACCAAAGCATCTTCATGAGAAGATATTTTTGCCATCTTCA-TTGTAGTATCCACC
AhMITE1_61   TGT-----TTAAAGTGTGGCTAAATAA---ATAAACCACACTTTTAACCAAAGCATCTTCATGAGAAGATATTTTTGCCATCTTCA-TTGTAGTATCCACC
AhMITE1_62   TATAAATATCAAAATCTAACCATACAATCCATCATCCAAAGGTCAAAAAGAAATATACTCATATGAAGACAATTATGCCATCTTCA-TTGTAGTATCCACC
AhMITE1_63   TGT-----TTAAAGTGTGGCTAAATAA---ATAAACCACACTTTTAACCAAAGCATCTTCATGAGAAGATATTTTTGCCATCTTCA-TTGTAGTATCCACC
AhMITE1_64   TGT-----TTAAAGTGTGGCTAAATAA---ATAAACCACACTTTTAACTAAAGCATCTTCATGAGAAGATATTTTTGCCATCTTCA-TTGTAGTATCCACC
AhMITE1_65   TGT-----TTAAAGTGTGGCTAAATAA---ATAAACCACACTTTTAACCAAAGCATCTTCATGAGAAGATATTTTTGCCATCTTCA-TTGTAGTATCCACC
AhMITE1_66   TGT-----TTAAAGTGTGGCTAAATAA---ATAAACCACACTTTTAACTAAAGCATCTTCATGAGAAGATATTTTTGCCATCTTCA-TTGTAGTATCCACC
AhMITE1_67   TGT-----TTAAAGTGTGGCTAAATAA---ATAAACCACACTTTTAACTAAAGCATCTTCATGAGAAGATATTTTTGCCATCTTCA-TTGTAGTATCCACC
AhMITE1_68   TGT-----TTAAAGTGTGGCTAAATAA---ATAAACCACACTTTTAACCAAAGCATCTTCATAAGAAGATATTTTTGCCATCTTCA-TAGTAGTATCCACC
AhMITE1_69   TGT-----TTAAAGTGTGGCTAAATAA---ATAAACCACACTTTTAATCAAAGCATCTTTATAAGAAGATATTTTTGCCATCTTCA-TTGTAGTATCCACC
AhMITE1_70   TGT-----TTAAAGTGTGGCTAAATAA---ATAAACCACACTTTTAACTAAAGCATCTTCATGAGAAGATATTTTTGCCATCTTCA-TTGTAGTATCCACC
AhMITE1_71   TGT-----TTAAAGTGTGGCTAAATAA---ATAAACTACACTTTTAACCAAAGCATCTTCATAAGAAGATATTTTTGCCATCTTCA-TTGTAGTATCCACC
AhMITE1_72   TGT-----TTAAAGTGTGGCTAAATAA---ATAAACCACACTTTTAACCAAAGCATATTTATGAGAAGATATTTTAGCCATCTTCA-TTGTAGTATCCACC
AhMITE1_73   TGT-----TTAAAGTGTGGCTAAATAA---ATAAACCACACTTTTAACCAAAGCATCTTCATGAGAAGATATTTTTGCCATCTTCA-TTGTAGTATCCACC
AhMITE1_74   TGT-----TTAAAGTGTGGCTAAATAA---ATAAACCACACTTTTAACTAAAGCATCTTCATGAGAAGATATTTTTGCCATCTTCA-TTGTAGTATCCACC
AhMITE1_75   TGT-----TTAAAGTGTGGCTAAATAA---ATAAACCACACTTTTAACTAAAGCATCTTCATGAGAAGATATTTTTGCCATCTTCA-TTGTAGTATCCACC
AhMITE1_76   TGT-----TTAAAGTGTGGCTAAATAA---ATAAACCACACTTTTAACCAAAGCATCTTCATGAGAAGATATTTTTGCCATCTTCA-TTGTAGTATCCACC
AhMITE1_77   TGT-----TTAAAGTGTGGCTAAATAA---ATAAACCACACTTTTAACAAGAGCATCTTCATGAGAAGATATTTTTGCCATCTTCA-TTGTAGTATCCACC
AhMITE1_78   TGT-----TTAAAGTGTAGCTAAATAA---ATAAACCACACTTTTAACCAAAGTATCTTCATGAGAAGATATTTTTGCCATCTTCA-TTGTAGTATCCACC
AhMITE1_79   TAT-----TTAAAGTGTGGCTAAATAA---ATAAACTACACTTTTAACCAAAGCATCTTCATAAGAAGATGTTTTTGCTATCTTCA-TTGTAGTATCCACC
AhMITE1_80   TAT-----TTAAAGTGTGGCTAGATAA---ATAAACCACACTTTTAACCAAAACAT-TTCATGAGAAGATATTTTTGCCATCTTCA-TTGTAGTATCTACC
AhMITE1_81   TGT-----TTAAAGTGTGGCTAAATAA---ATAAACCACACTTTTAACTAAAGCATCTTTATGAGAAGATATTTTTGCCATCGTCA-TTATAATAACTACC
AhMITE1_82   TGT-----TTAAAGTGTGGCTAAATAA---ATAAACCACACTTTTAACCAAAGCATCTTCATAAGAAGATATTTTTGCCATCTTCA-TAGTAGTATCCACC
AhMITE1_83   TGT-----TTAAAGTGTGGCTAAATAA---ATAAACCACACTTTTAACCAAAGCATCTCCATGAGAAGATATTTTTGCCATCTTCA-TTGTAGTATCCACC
AhMITE1_84   TGT-----TTAAAGTGTGGCTAAATAA---ATAAACCACACTTTTAACCAAAGCATCTTCATGAGAAGATATTTTTGCCATCTTCA-TTGTAGTATCCACC
AhMITE1_85   TGT-----TTAAAGTGTGGCTAAATAA---ATAAACCACACTTTTAACCAAAGCATCTTCATGAGAAGATATTTTTGCCATCTTCA-TTGTAGTATCCACC
AhMITE1_86   TGT-----TTAAAGTGTGGCTAAATAA---ATAAACCACACTTTTAACCAAAGCATCTTCATGAGAAGATATTTTTGCCATCTTCA-TTGTAGTATCCACC
AhMITE1_87   TGT-----TTAAAGTGTGGCTAAATAA---ATAAACCACACTTTTAACCAAAGCATCTTCATGAGAAGATATTTTTGCCATCTTCA-TTGTAGTATCCACC
AhMITE1_88   TGT-----TTAAAGTGTGGCTAAATAA---ATAAACCACACTTTTAACTAAAGCATCTTCATGAGAAGATATTTTTGCCATCTTCA-TTGTAGTATCCACC
AhMITE1_89   TGT-----TTAAAGTGTGGCTAAATAA---ATAAACCACACTTTTAACTAAAGCATCTTCATGAGAAGATATTTTTGCCATCTTCA-TTGTAGTATCCACC
AhMITE1_90   TGT-----TTAAAGTGTGGCTAAATAA---ATAAACCACACTTTTAACCAAAGCATCTTCATGAGAAGATATTTTTGCCATCTTCA-TTGTAGTATCCACC
AhMITE1_91   TGT-----TTAAAGTGTGGCTAAATAA---ATAAACCACACTTTTAACCAAAGCATTTTCATGAGAAGATATTTTTACCATCTTCA-TTGTAGTATCCACC
AhMITE1_92   TGT-----TTAAAATGGGGCTAAATAA---ATAAACCACACTTTTAACCAAAGCATCTTCATGAAAAGATATTTTTGTCATCTTCA-TTGTAGTATCCACC
AhMITE1_93   TGT-----TTAAAATGTGGCTAAATAA---ATAAACCACACTTTTAACCAAAGCATCTTCATGAGAATATATTTTTGCCATTTTCA-TTGTAGTATCCACC
AhMITE1_94   TGT-----TTAAAGTGTGGCTAAATAA---ATAAACCACACTTTTAACCAAAGCATCTTCATGAGAAGATATTTTTGCCATCTTCA-TTGTAGTATCCACC
AhMITE1_95   TGT-----TTAAAGTGTGGCTAAATAA---ATAAACCACACTTTTAACCAAAGCATCTTCATGAGAAGATATTTTTGCCATCTTCA-TTGTAGTATCCACC
AhMITE1_96   TATAAATATCAAAATCTAACCATACAATCCATCATCCAAAGGTCAAAAAGAAATATACTCATATGAAGACAATTATGCCATCTTCA-TTGTAGTATCCACC
AhMITE1_97   TGT-----TTAAAGTGTGGCTAAATAA---ATAAACCACACTTTTAACCAAAGCATCTTCATGAGAAGATATTTTTGCCATCTTCA-TTGTAGTATCCACC
AhMITE1_98   TGT-----TTAAAGTGTGGCTAAATAA---ATAAACCACACTTTTAACCAAAGCATCTTCATGAGAAGATATTTTTGCCATCTTCA-TTGTAGTATCCACC
AhMITE1_99   TGT-----TTAAAGTGTGGCTAAATAA---ATAAACCACACTTTTAACCAAAGCATCTTCATGAGAAGATATTTTTGCCATCTTCA-TTGTAGTATCCACC
AhMITE1_100  TGT-----TTAAAGTGTGGCTAAATAA---ATAAACCACACTTTTAACCAAAGCATCTTCATGAGAAGATATTTTTGCCATCTTCA-TTGTAGTATCCACC
```

```
                                    130       140       150       160       170       180       190       200       210       220
AhMITE1_101  TGT-----TTAAAGTGTGGCTAAATAA---ATAAACCACACTTTTAACCAAAGCATCTTCATGAGAAGATATTTTTGCCATCTTCA-TTGTAGTATCCACC
AhMITE1_102  TGT-----TTAAAGTGTGGCTAAATAA---ATAAACCACACTTTTAACCAAAGCATCTTCATGAGAAGATATTTTTGCCATCTTCA-TTGTAGTATCCACC
AhMITE1_103  TGT-----TTAAAGTGTGGCTAAATAA---ATAAACCACACTTTTAACCAAAGCATCTTCATGAGAAGATATTTTTGCCATCTTCA-TTGTAGTATCCACC
AhMITE1_104  TGT-----TTAAAATGTGGCTAAATAA---ATAAACCACACTTTTAAC-AAAGCATCTTCATGAGAAGATATTTTTGCCATCTTCA-TTGTAGTATCCACC
AhMITE1_105  TGT-----TTAAAGTGTGGCTAAATAA---ATAAACCACACTTTTAACCAAAGCATCTTCATGAGAAAATATTTTTACCATCTTTA-TTGTAGTATCCACC
AhMITE1_106  TGT-----TTAAAATGTGGCTAAATAA---ATAAACCACACTTTTAACCAAAGCATCTTCATGAGAAGATATTTTTGTCATCTTCA-TTGTAGTATCCACC
AhMITE1_107  TGT-----TTAAAGTGTGGCTAAATAA---ATAAACCACACTTTTAACCAAAGCATCTTCATGAGAAGATATTTTTGCCATCTTCA-TTGTAGTATCCACC
AhMITE1_108  TGT-----TTAAAGTGTGGCTAAATAA---ATAAACCACACTTTTAACCAAAGCATCTTCATGAGAAGATATTTTTGCCATCTTCA-TTGTAGTATCCACC
AhMITE1_109  TGT-----TTAAAGTGTGGCTAAATAA---ATAAACCACACTTTTAACCAAAGCATCTTCATGAGAAGATATTTTTGCCATCTTCA-TTGTAGTATCCACC
AhMITE1_110  TGT-----TTAAAGTGTGGCTAAATAA---ATAAACCACACTTTTAACCAAAGCATCTTCATGAGAAGATATTTTTGCCATCTTCA-TTGTAGTATCCACC
AhMITE1_111  TGT-----TTAAAGTGTGGCTAAATAA---ATAAACCACACTTTTAACTAAAGCATCTTCATGAGAAGATATTTTTGCCATCTTCA-TTGTAGTATCCACC
AhMITE1_112  TGT-----TTAAAGTGTGGCTAAATAA---ATAAACCACACTTTTAACCAAAGCATCTTCATGAGAAGATATTTTTGTCATCTTCA-TTGTAGTATCCACC
AhMITE1_113  TAT-----TTAAAGTGTGACTAGATAA---ATAAACTACACTTTTAACCAAAGCATCTTCATGAGAAGATATTTTTGCCATCTTCA-TTATAGTATCCACC
AhMITE1_114  TGT-----TTAAAGTGTGGCTAAATAA---ATAAACCACACTTTTAATCAAAGCATCTTCATGAAAAGATAGTTTTATCATCTTCA-TTGTAGTATCCACC
AhMITE1_115  TGT-----TTAAAGTGTGGCTAAATAA---ATAAACCACACTTTTAACCAAAGCATCTTCATGAGAAGATATTTTTGCCATCTTCA-TTGTAGTATCCACC
AhMITE1_116  TGT-----TTAAAGTGTGGCTAAATAA---ATAAACCACACTTTTAACCAAAGCATCTTCATGAGAAGATATTTTTGCCATCTTCA-TTGTAGTATCCACC
AhMITE1_117  TGT-----TTAAAGTGTGGCTAAATAA---ATAAACCACACTTTTAACCAAAGCATCTTCATGAGAAGATATTTTTGCCATCTTCA-TTGTAGTATCCACC
AhMITE1_118  TGT-----TTAAAGTGTGGCTAAATAA---ATAAACCACACTTTTAACCAAAGCATCTTCATGAGAAGATATTTTTGCCATCTTCA-TTGTAGTATCCACC
AhMITE1_119  TGT-----TTAAAGTGTGGCTAAATAA---ATAAACTACACTTTTAACCAAAGCATCTTCATGAGAAGATATTTTTGCCATCTTCA-TTGTAGTATCCACC
AhMITE1_120  TGT-----TTAAAGTGTGGCTAAATAA---ATAAACTACACTTTTAACCAAAGCATCTTCATGAGAAGATATTTTTGCCATCTTCA-TTGTAGTATCCACC
AhMITE1_121  TGT-----TTAAAGTGTGGCTAAATAA---ATAAACCACACTTTTAACCAAAGTATCTTTATGAGAAGATATTTTTGCCATCTTCA-TTGTAGTATCCACC
AhMITE1_122  TGT-----TTAAAGTGTGGCTAAATAA---ATAAACCACACTTTTAACCAAAGCATCTTCATGAGAAGATATTTTTGCCATCTTCA-TTGTAGTATCCACC
AhMITE1_123  TGT-----TTAAAGTGTGACTAAATAA---ATAAACCACACTTTTAACCAAAGCATCTTCATAAGAAAATATTTTTGCCATCTTCA-TTATAGTATCCACC
AhMITE1_124  T-T-----TTAAAGTGTGGCTAAATAA---ATAAACCACACTTTTAACCAAAGCTTCTTCATGAAAAGATATTTTTATCATCTTCA-TTGTAGTATCCACA
AhMITE1_125  TGT-----TTAAAGTGTGGCTAAATAA---ATAAACCACACTTTTAACCAAAGCATCTTCATGAGAAGATATTTTTGCCATCTTCA-TTGTAGTATCCACC
AhMITE1_126  TGT-----TTAAAGTGTGGCTAAATAA---ATAAACCACACTTTTAACCAAAGCATCTTCATGAGAAGATATTTTTGCCATCTTCA-TTGTAGTATCCACC
AhMITE1_127  TGT-----TTAAAGTGTGGCTAAATAA---ATAAACCACACTTTTAACTAAAGCATCTTCATGAGAAGATATTTTTGCCATCTTCA-TTGTAGTATCCACC
AhMITE1_128  TGT-----TTAAAGTGTGGCTAAATAA---ATAAACCACACTTTTAACCAAAGCATCTTCATAAGAAGATATTTTTGCCATCTTCA-TAGTAGTATCCACC
AhMITE1_129  --G-----TTAAAGTGTGGCTAAATAA---ATAAACCACACTTTTAACCAAAGCATCTTCATAAGAAGATATTTTTGCCATCTTCA--AGTAGTATCCACC
AhMITE1_130  TGT-----TTAAAATGTGGCTAAATAA---ATAAACCACACTTTTAACCAAAGCATCTTCATGAGAAAATATTTTTGCCATCTTCA-TTGTAATATCCACC
AhMITE1_131  TGT-----TTAAAGTGTGGCTAAATAA---ATAAACCACACTTTTAACCAAAGCATCTTCATGAGAAGATATTTTTGCCATCTTCA-TTGTAGTATCCACC
AhMITE1_132  TGT-----TTAAAGTGTGGCTAAATAA---ATAAACCACACTTTTAACCAAAGCATCTTCATGAGAAGATATTTTTGCCATCTTCA-TTGTAGTATCCACC
AhMITE1_133  TGT-----TTAAAGTGTGGCTAAATAA---ATAAACCACACTTTTAACCAAAGCATCTTCATGAGAAGATATTTTTGCCATCTTCA-TTGTAGTATCCACC
AhMITE1_134  TGT-----TTAAAGTGTGGCTAAATAA---ATAAACCACACTTTTAACCAAAGCATCTTCATGAGAAGATATTTTTGCCATCTTCA-TTGTAATATCCACC
AhMITE1_135  TGT-----TTAAAGTGTGGCTAAATAA---ATAAACCACACTTTTAACCAAAGCATCTTCATGAGAAGATATTTTTGCCATCTTCA-TTGTAGTATCCACC
AhMITE1_136  TGT-----TTAAAGTGTGGCTAAATAA---ATAAACCACACTTTTAACTAAAGCATCTTCATGAGAAGATATTTTTGCCATCTTCA-TTGTAGTATCCACC
AhMITE1_137  TGT-----TTAAAGTGTGGCTAAATAA---ATAAACCACACTTTTAACTAAAGCATCTTCATGAGAAGATATTTTTGCCATCTTCA-TTGTAGTATCCACC
AhMITE1_138  TGT-----TTAAAGTGTGGCTAAATAA---ATAAACCACACTTTTAACCAAAGTATCTTCATGAGAAGATATTCTTGCCATCTTCA-TTGTAGTATCCACC
AhMITE1_139  TGT-----TTAAAGTGTGGCTAAATAA---ATAAACCACACTTTTAACCAAAGCATCTTCATGAGAAGATATTTTTGCCATCTTCA-TTGTAGTATCCACC
AhMITE1_140  TGT-----TTAAAGTGTGGCTAAATAA---ATAAACCACACTTTTAACCAAAGCATCTTCATGAGAAGATATTTTTGCCATCTTCA-TTGTAGTATCCACC
AhMITE1_141  TGT-----TTAAAGTGTGGCTAAATAA---ATAAACCACACTTTTAACCAAAGCATCTTCATGAGAAGATATTTTTGCCATCTTCA-TTGTAGTATCCACC
AhMITE1_142  TAT-----TTAAAGTGTGACTAAATAA---ATAAACCACACTTTTAACCAAAGCATCTTCATGAGAAGATATTTTTGCCATCTTCA-TTGTAGTATCCACC
```

Supplementary Fig. 4 Sequence alignment of 142 *AhMITE1* members. The dotted lines represent gaps.
