# Supplementary figures and images for: Identification and characterization of transposable element AhMITE1 in the genomes of cultivated and two wild peanuts
Source: BMC Genomics. 2022 Jul 11;23:500. doi: 10.1186/s12864-022-08732-0 (PMC9277781; doi:10.1186/s12864-022-08732-0)

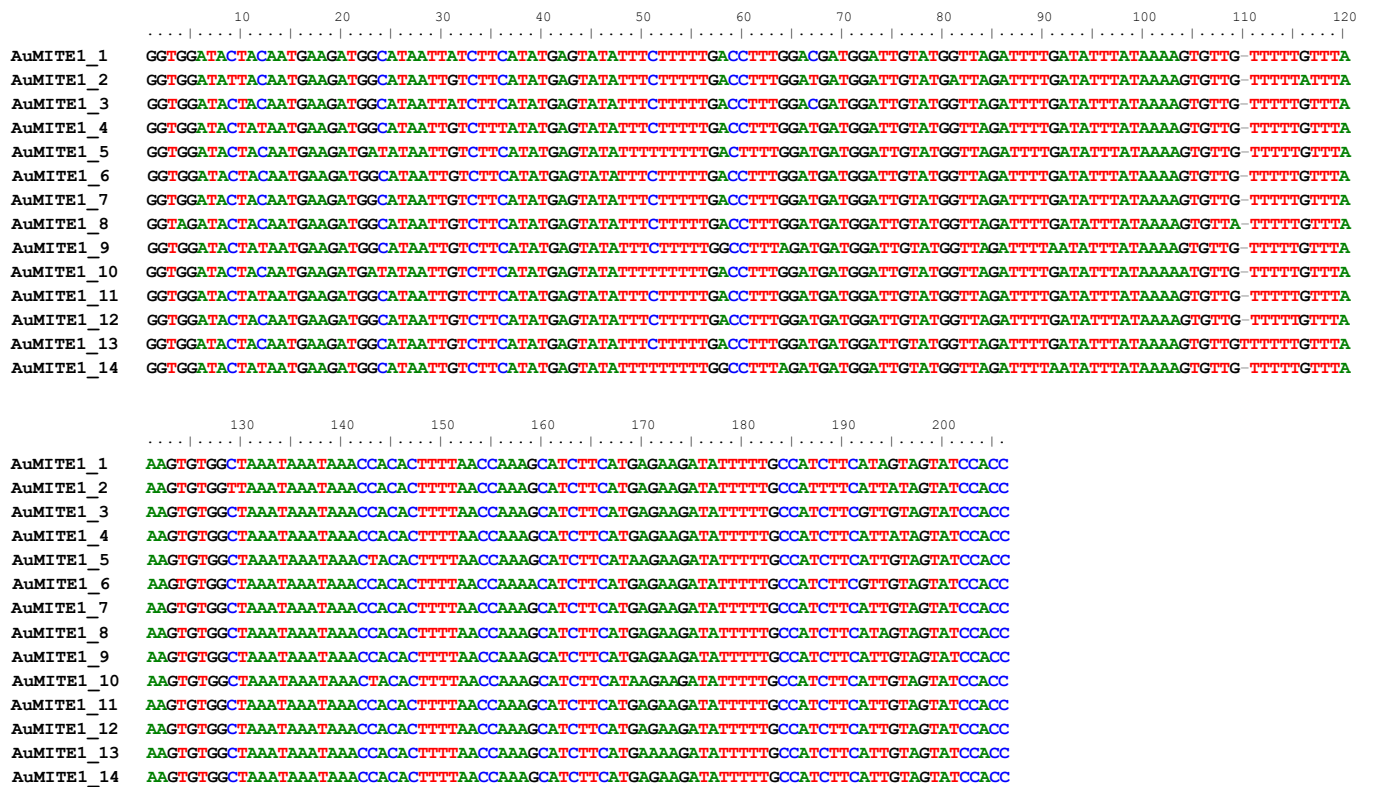

**Supplementary Fig. 5** Sequence alignment of 14 *AuMITE1* members. The dotted lines represent gaps.

Supplement: Supplementary file 5 — Additional file 5: Supplementary fig 5. [file 12864_2022_8732_MOESM5_ESM.pdf]

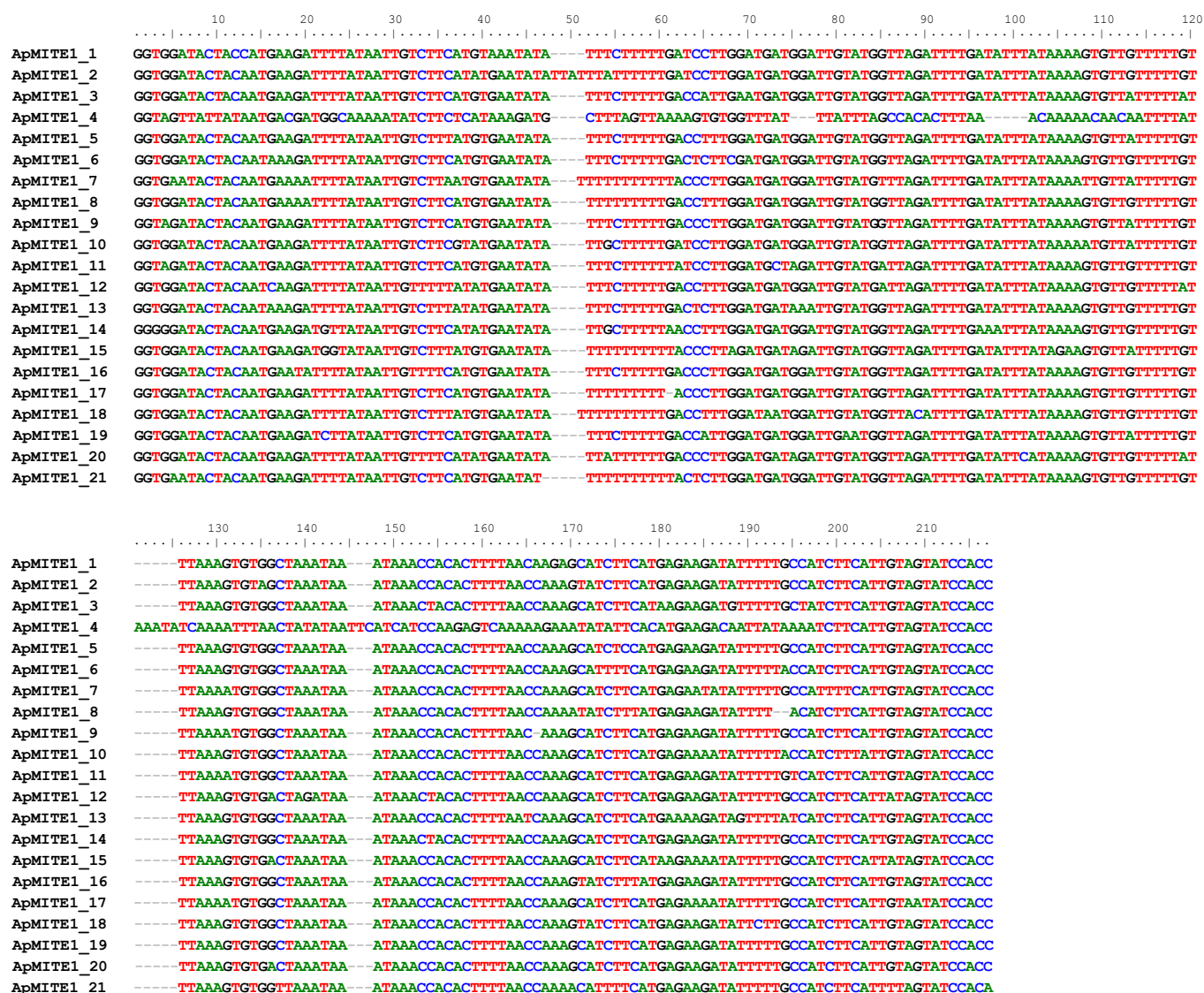

**Supplementary Fig. 6** Sequence alignment of 21 *ApMITE1* members. The dotted lines represent gaps.

Supplement: Supplementary file 6 — Additional file 6: Supplementary fig 6. [file 12864_2022_8732_MOESM6_ESM.pdf]
