# Supplementary material for: Identification and characterization of transposable element AhMITE1 in the genomes of cultivated and two wild peanuts
Source: BMC Genomics. 2022 Jul 11;23:500. doi: 10.1186/s12864-022-08732-0 (PMC9277781; doi:10.1186/s12864-022-08732-0)
Supplement: Supplementary file 7 — Additional file 7: Supplementary fig 7. [file 12864_2022_8732_MOESM7_ESM.pdf]

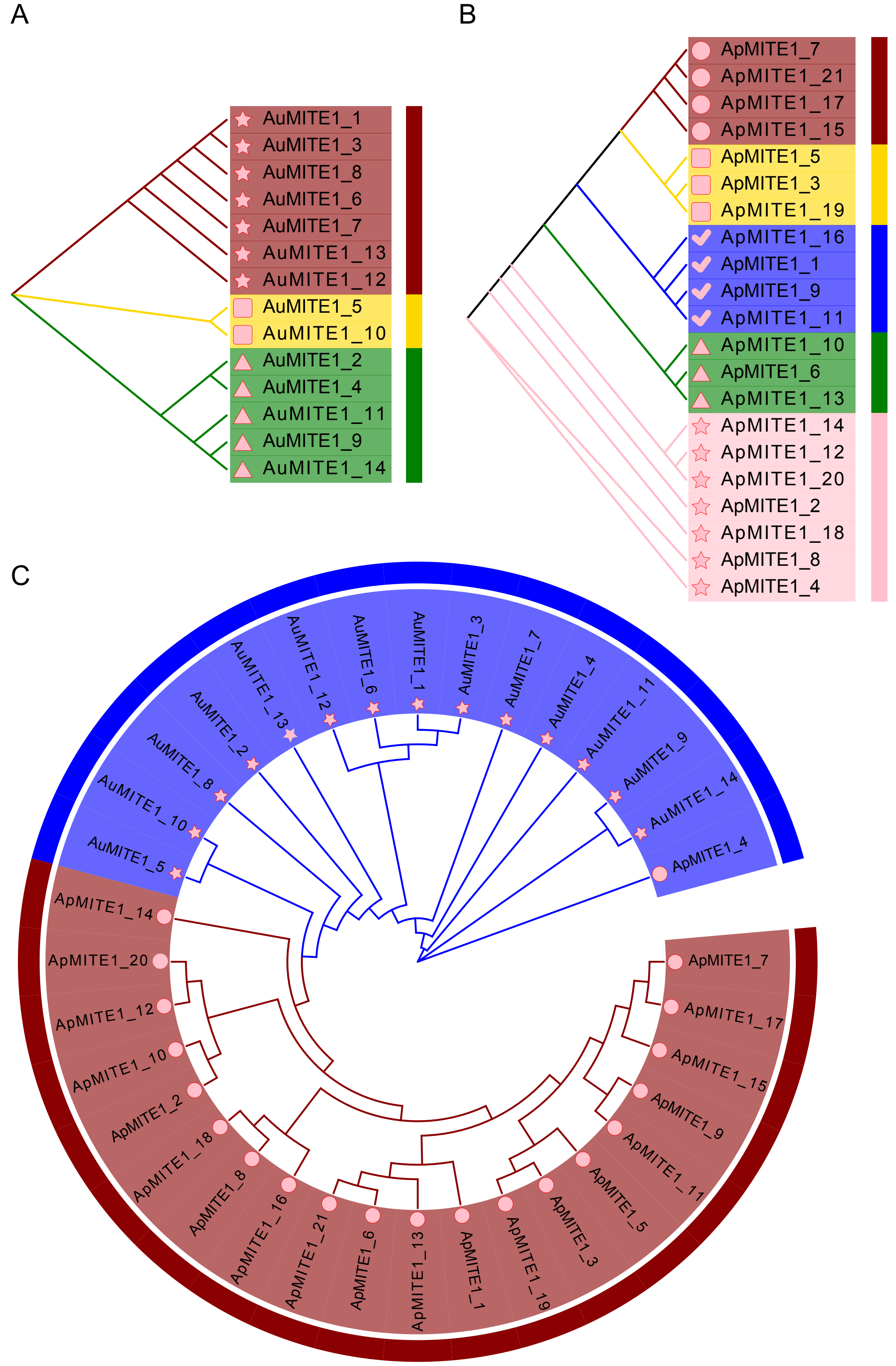

Supplementary Fig. 7 Phylogenetic tree comprising 14 *AuMITE1* (A) and 21 *ApMITE1* (B) from *A. duranensis* and *A. ipaensis*, respectively. (A) Red, gold and green indicate Clade I, Clade II and Clade III, respectively. (B) Red, gold, blue, green and pink indicate Clade I, Clade II, Clade III, Clade IV and Clade V, respectively. (C) Phylogenetic tree comprising *AuMITE1* and *ApMITE1* elements. Red and blue indicate Clade I and Clade II, respectively.
