# Supplementary material for: Identification and characterization of transposable element AhMITE1 in the genomes of cultivated and two wild peanuts
Source: BMC Genomics. 2022 Jul 11;23:500. doi: 10.1186/s12864-022-08732-0 (PMC9277781; doi:10.1186/s12864-022-08732-0)
Supplement: Supplementary file 8 — Additional file 8: Supplementary fig 8. [file 12864_2022_8732_MOESM8_ESM.pdf]

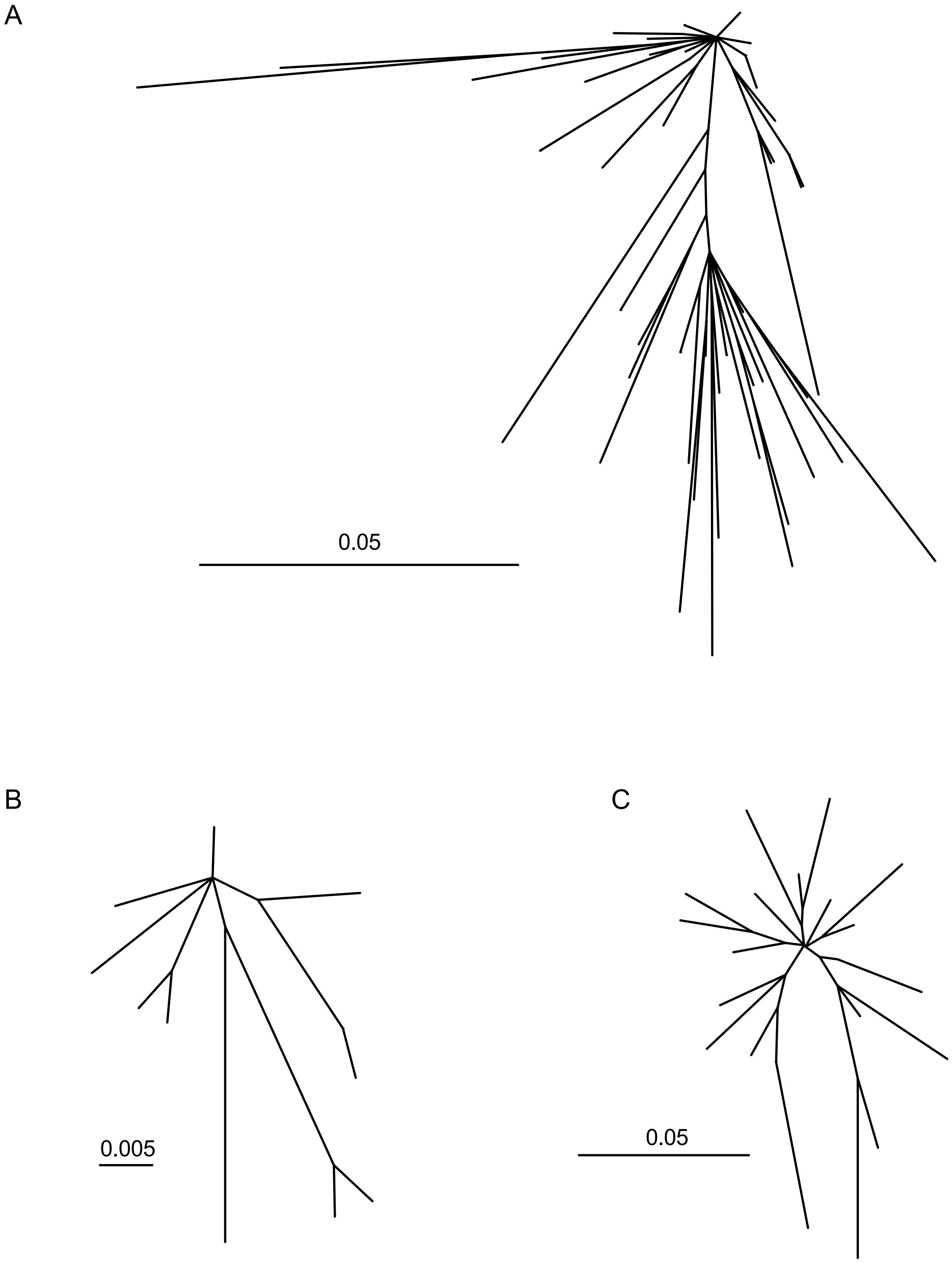

Supplementary Fig. 8 Phylogenetic tree of *AhMITE1* (A), *AuMITE1* (B) and *ApMITE1* (C) families with unimodal distribution of pairwise nucleotide diversity.The star shape tree suggests one round of amplification burst.
