# Supplementary material for: Identification and characterization of transposable element AhMITE1 in the genomes of cultivated and two wild peanuts
Source: BMC Genomics. 2022 Jul 11;23:500. doi: 10.1186/s12864-022-08732-0 (PMC9277781; doi:10.1186/s12864-022-08732-0)
Supplement: Supplementary file 9 — Additional file 9: Supplementary fig 9. [file 12864_2022_8732_MOESM9_ESM.pdf]

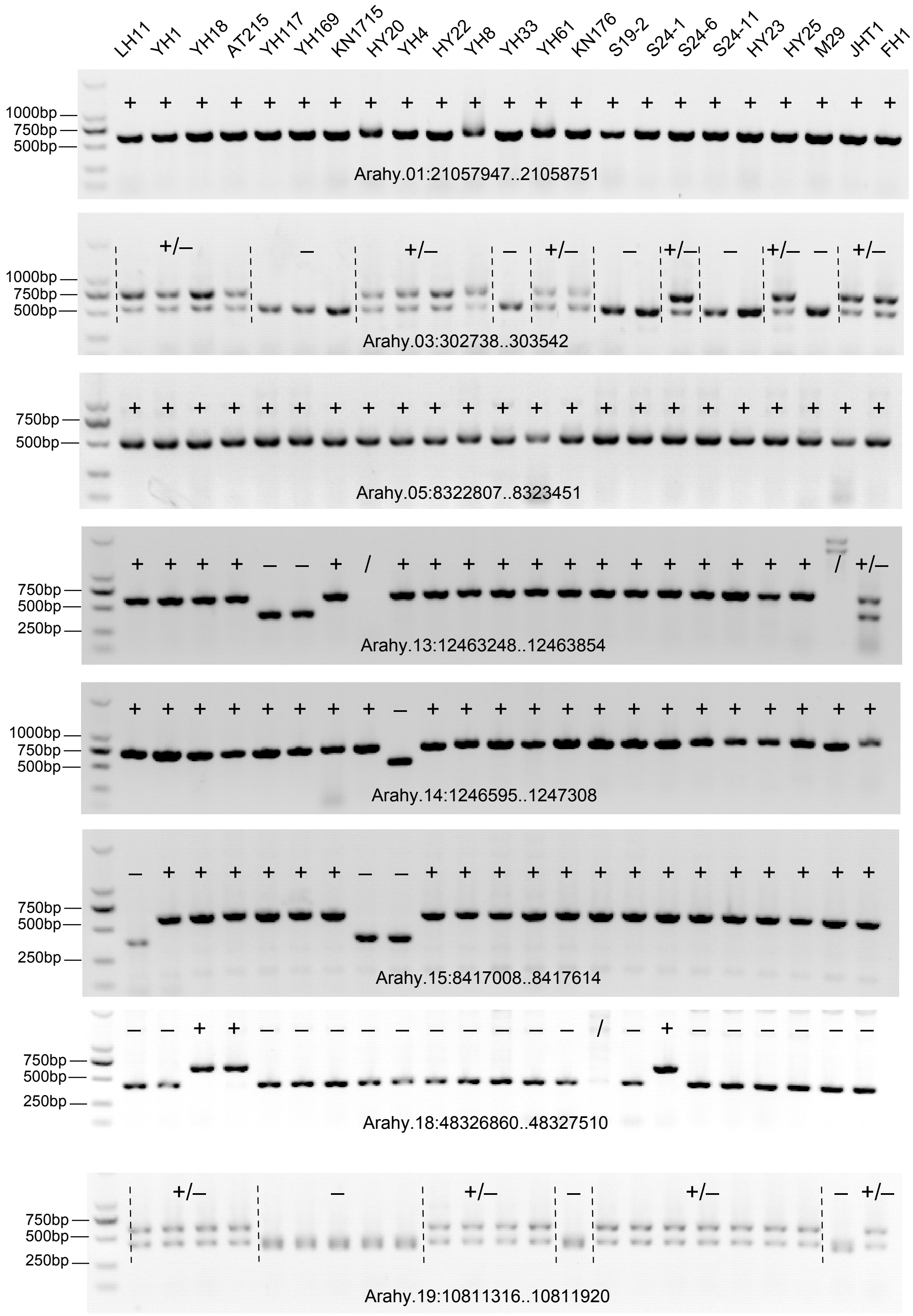

Supplementary Fig. 9 Validation of the presence of an *AhMITE1* insertion identified using PCR analysis in different varieties. Validation of the presence of an *AhMITE1* insertion using locus -specific PCR analysis. Plus and minus represent the presence and absence of the *AhMITE1* insertion, respectively.
