# Supplementary material for: Identification and characterization of transposable element AhMITE1 in the genomes of cultivated and two wild peanuts
Source: BMC Genomics. 2022 Jul 11;23:500. doi: 10.1186/s12864-022-08732-0 (PMC9277781; doi:10.1186/s12864-022-08732-0)
Supplement: Supplementary file 10 — Additional file 10: Supplementary fig 10. [file 12864_2022_8732_MOESM10_ESM.pdf]

A

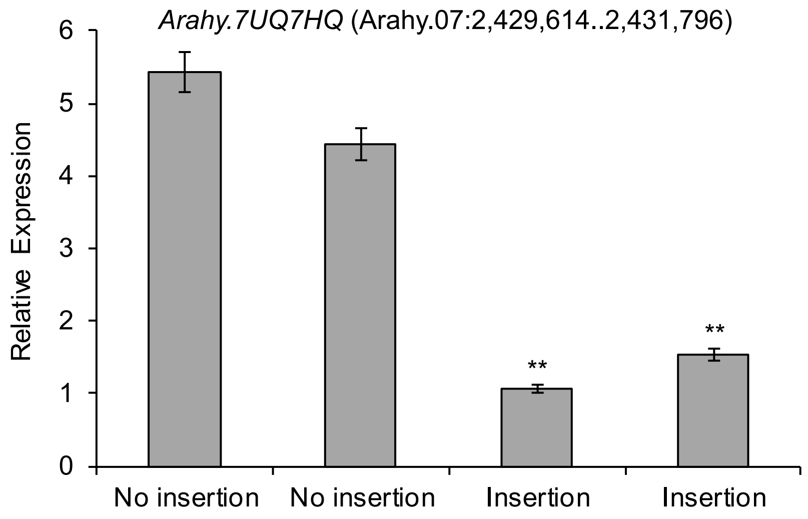

B

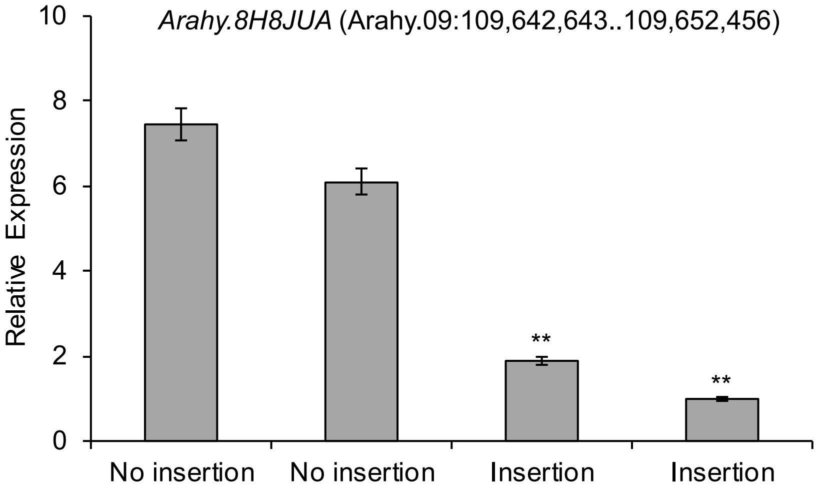

Supplementary Fig. S10 Relative expression of genes *Arahy.7UQ7HQ* and *Arahy.8H8JUA* with or without *AhMITE1* insertion. The statistical significance was set at $P < 0.05$ based on a two-tailed Student's t test, $^{**}P < 0.01$.
